# A scoping review of echocardiographic and lung ultrasound biomarkers of bronchopulmonary dysplasia in preterm infants

**DOI:** 10.3389/fped.2023.1067323

**Published:** 2023-02-10

**Authors:** Silvia Martini, Iuri Corsini, Luigi Corvaglia, Pradeep Suryawanshi, Belinda Chan, Yogen Singh

**Affiliations:** ^1^ Neonatal Intensive Care Unit, IRCCS AOUBO, Department of Medical and Surgical Sciences, University of Bologna, Bologna, Italy; ^2^ Division of Neonatology, Careggi University Hospital of Florence, Florence, Italy; ^3^ Department of Neonatology, Bharati Vidyapeeth University Medical College, Pune, India; ^4^ Division of Neonatology, University of Utah, Salt Lake City, UT, United States; ^5^ Department of Pediatrics – Division of Neonatology, Loma Linda University School of Medicine, Loma linda, CA, United States; ^6^ Neonatology/Pediatric Cardiology, Cambridge University Hospitals, Cambridge, United Kingdom

**Keywords:** bronchopulmonary dysplasia, pulmonary hypertension, echocardiography, lung ultrasound, preterm infants

## Abstract

Despite recent improvements in neonatal care, moderate to severe bronchopulmonary dysplasia (BPD) is still associated with high mortality and with an increased risk of developing pulmonary hypertension (PH). This scoping review provides an updated overview of echocardiographic and lung ultrasound biomarkers associated with BPD and PH, and the parameters that may prognosticate their development and severity, which could be clinically helpful to undertake preventive strategies. A literature search for published clinical studies was conducted in PubMed using MeSH terms, free-text words, and their combinations obtained through appropriate Boolean operators. It was found that the echocardiography biomarkers for BPD, and especially those assessing right ventricular function, are reflective of the high pulmonary vascular resistance and PH, indicating a strong interplay between heart and lung pathophysiology; however, early assessment (e.g., during the first 1–2 weeks of life) may not successfully predict later BPD development. Lung ultrasound indicating poor lung aeration at day 7 after birth has been reported to be highly predictive of later development of BPD at 36 weeks' postmenstrual age. Evidence of PH in BPD infants increases risk of mortality and long-term PH; hence, routine PH surveillance in all at risk preterm infants at 36 weeks, including an echocardiographic assessment, may provide useful information. Progress has been made in identifying the echocardiographic parameters on day 7 and 14 to predict later development of pulmonary hypertension. More studies on sonographic markers, and especially on echocardiographic parameters, are needed for the validation of the currently proposed parameters and the timing of assessment before recommendations can be made for the routine clinical practice.

## 1. Introduction

The term bronchopulmonary dysplasia (BPD) defines a chronic developmental respiratory disease diagnosed at 36 weeks gestational age (GA) in the extremely preterm infants, resulting from combination of multiple antenatal and postnatal risk factors. While intrauterine growth restriction, chorioamnionitis and maternal smoking are established antenatal risk factors, postnatal triggers include surfactant deficiency, ventilation-induced lung injury (VILI), oxygen toxicity, lung inflammation, and inadequate nutrition to support lung growth (1). The significant advances in neonatal care occurred over the past decades, such as antenatal steroids and exogenous surfactant replacement, have decreased morbidity and mortality in extremely preterm infants. However, the incidence of BPD has remained substantially unchanged, possibly due to increased survival of the extremely preterm infants, especially those around peri-viable period (22–24 weeks) (1). We also recognize that pathophysiology of BPD has evolved with more extremely preterm survival and change in the management strategies in the NICU. In the pre-surfactant era, the BPD is predominately resulted from VILI leading to marked regional inflammation. The current “new” BPD features are primarily characterized by abnormally reduced number and structurally simplified alveoli, and by dysmorphic pulmonary arteries (2, 3).

Insights from experimental models indicate that “new” BPD results from disruption of physiological interactions between pulmonary angiogenesis and alveolarization. The deranged pathways include: (i) the nitric oxide/guanosine monophosphate cyclic system, with antiproliferative effects, (ii) the prostaglandin and arachidonic acid pathway with vasodilative effects, and (iii) the endothelin pathway with vasoconstrictor and remodelling effects (4). In turn, abnormal development of alveoli and lung microvasculature contribute to determine specific BPD phenotypes that can partially overlap lung parenchymal disease, airway disease, and pulmonary vascular disease (PVD) (5). The haemodynamic presentation of the increased pulmonary vascular resistances (PVR) from BPD associated PVD can range from right ventricular dysfunction to pulmonary hypertension (PH), which is burdened by high mortality rates and poor long-term outcomes (6).

Due to the strong developmental interaction between lung and cardiovascular systems, and a strong association between BPD and PH, echocardiography has been increasingly used in preterm infants with BPD to evaluate for PH. Many echocardiographic parameters associated with BPD and its severity are predominantly related to PVD, PH, and altered cardiac haemodynamics.

Lung ultrasound is an imaging modality that has gained increasing popularity to study lung disease in neonatal population (7). Its applications in neonates include diagnosis of lung diseases and their severity, prediction of the need for surfactant, and readiness for extubation. Recent studies on sonographic biomarkers have shown promising results in predicting BPD. However, the majority of these studies were published in recent years, some even have seemingly conflicting information and different levels of evidence. Understanding these sonographic biomarkers for BPD and PH are vital for optimising clinical management and prevention strategies.

The aims of this scoping review are: (1) to provide an updated overview of the available literature on the association between sonographic biomarkers and BPD and PH during first year after birth, (2) to critically review the current echocardiographic and lung ultrasound biomarkers associated with BPD and PH, and (3) to identify knowledge gaps in the available literature.

## 2. Methods

A literature search was conducted to identify clinical studies published before December 24, 2022 in PubMed (http://www.ncbi.nlm.nih.gov/pubmed/) that evaluated the association between specific sonographic biomarkers and BPD or BPD-related PH. Specific PubMed MeSH terms, free-text words, and their combinations obtained through the most proper Boolean operators were used for the literature search. In particular, the following strings were built up:

• (infant OR infant* OR neonate OR neonat* OR newborn OR newborn*) AND (preterm OR premature birth* OR Infant, Premature [MeSH]) AND ((bronchopulmonary dysplasia [MeSH] OR (BPD) OR (chronic lung disease)) AND (Echocardiography [MeSH] OR Echocardiography, Doppler [MeSH] OR (ECHO)).
• (infant OR infant* OR neonate OR neonat* OR newborn OR newborn*) AND (preterm OR premature birth* OR Infant, Premature [MeSH]) AND ((pulmonary hypertension [MeSH] OR (pulmonary vascular disease)) AND (Echocardiography [MeSH] OR Echocardiography, Doppler [MeSH] OR (ECHO))
• (infant OR infant* OR neonate OR neonat* OR newborn OR newborn*) AND (preterm OR premature birth* OR Infant, Premature [MeSH]) AND ((bronchopulmonary dysplasia [MeSH] OR (BPD) OR (chronic lung disease)) AND (lung ultrasound).
• (infant OR infant* OR neonate OR neonat* OR newborn OR newborn*) AND (preterm OR premature birth* OR Infant, Premature [MeSH]) AND ((pulmonary hypertension [MeSH]) OR (pulmonary vascular disease)) AND (lung ultrasound).

Bibliographic lists of the resulting papers were also checked for further literature implementation.

Book chapters, editorials and commentaries, consensus statements or opinion articles, meeting abstracts, reviews and meta-analyses, study protocols, *in vitro* or animal studies, studies on adults/pediatric cohorts, studies dealing with neonatal conditions other from BPD/PH (e.g., persistent pulmonary hypertension of the newborn) were excluded. Studies published in languages other than English were also excluded. In order to focus on short-term and medium-term findings, only follow-up studies assessing echocardiographic or lung ultrasound parameters at ≤1 year corrected age (CA) were included.

The echocardiographic and lung ultrasound biomarkers that have been investigated in the studies retrieved by the above searches are described below. Representative figures are also provided.

### 2.1. Echocardiographic parameters

#### 2.1.1. Tricuspid regurgitation jet velocity (TRJV) and right ventricular systolic pressure (RVSP)

**Figure 1:**
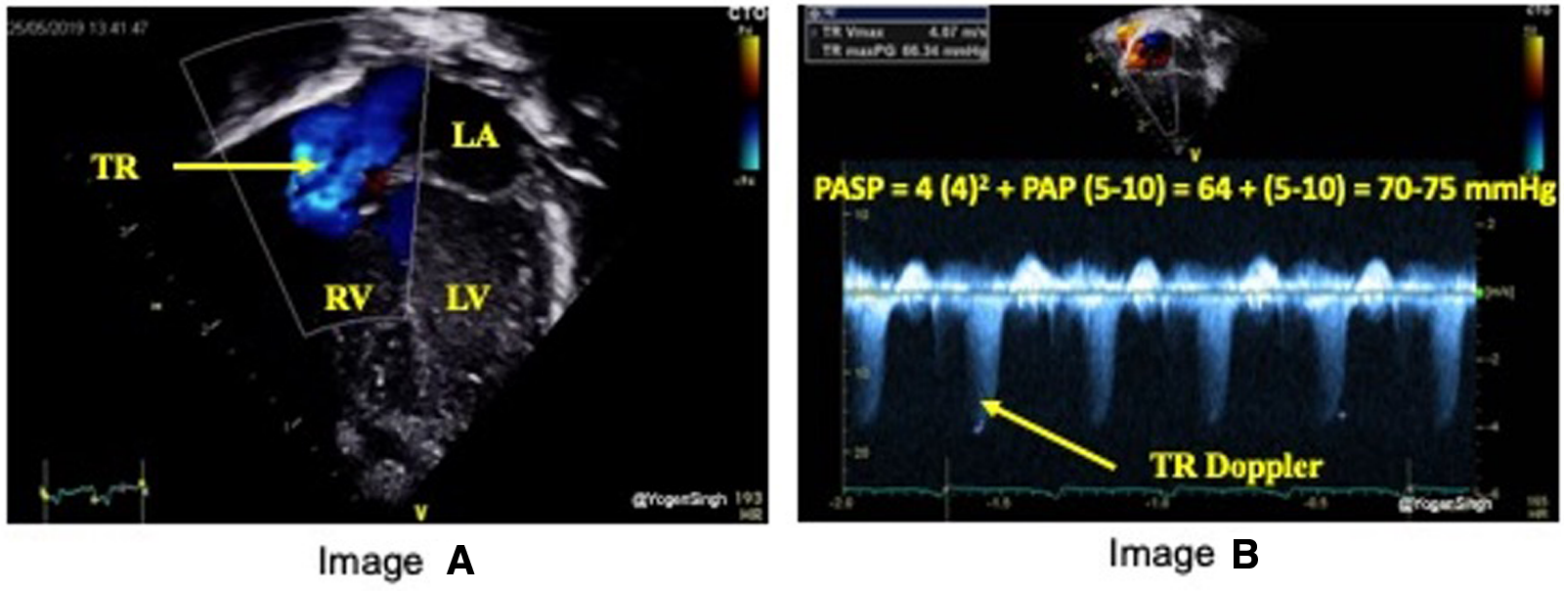
Estimation of pulmonary artery systolic pressure (PASP). Image “**A**” shows TR jet in apical 4 chamber view (A4C) and image “**B**” with Doppler assessment of tricuspid regurgitation (TR) – in this case a gradient of around 64 mmHg (TR jet velocity of 4 m/s) between right ventricle (RV) and right atrium (RA) suggesting PASP between 70 and 75 mmHg (64 + RA pressure of 5–10 mmHg).

TRJV can be assessed using continuous wave Doppler from multiple echocardiographic views: apical 4 chamber (Figure 1), parasternal long axis sweep, parasternal short axis, or subcostal four-chamber. The view allowing the best image quality and minimal angle of insonation should be preferred to sample TRJV, from which it is possible to derive pressure gradient between right ventricle and right atrium using the modified Bernoulli equation: = 4 × (TRJV)^2^. RVSP or pulmonary artery systolic pressure (PASP) can be estimated by adding right atrial pressure of 5–10 mmHg to pressure gradient measured by TRJV. New PH definition includes a mean PASP over 20 mmHg (8); however, it is applicable in infants aged 3 months or older. PH in infants under 3 months of age is not clearly defined and usually diagnosed on echocardiography with a TRJV over 2.8 m/s (PASP >36 mmHg) or other features of PH (*see PH section*) (9).

#### 2.1.2. Septal flattening and left ventricular eccentricity index (LV-EI)

**Figure 2:**
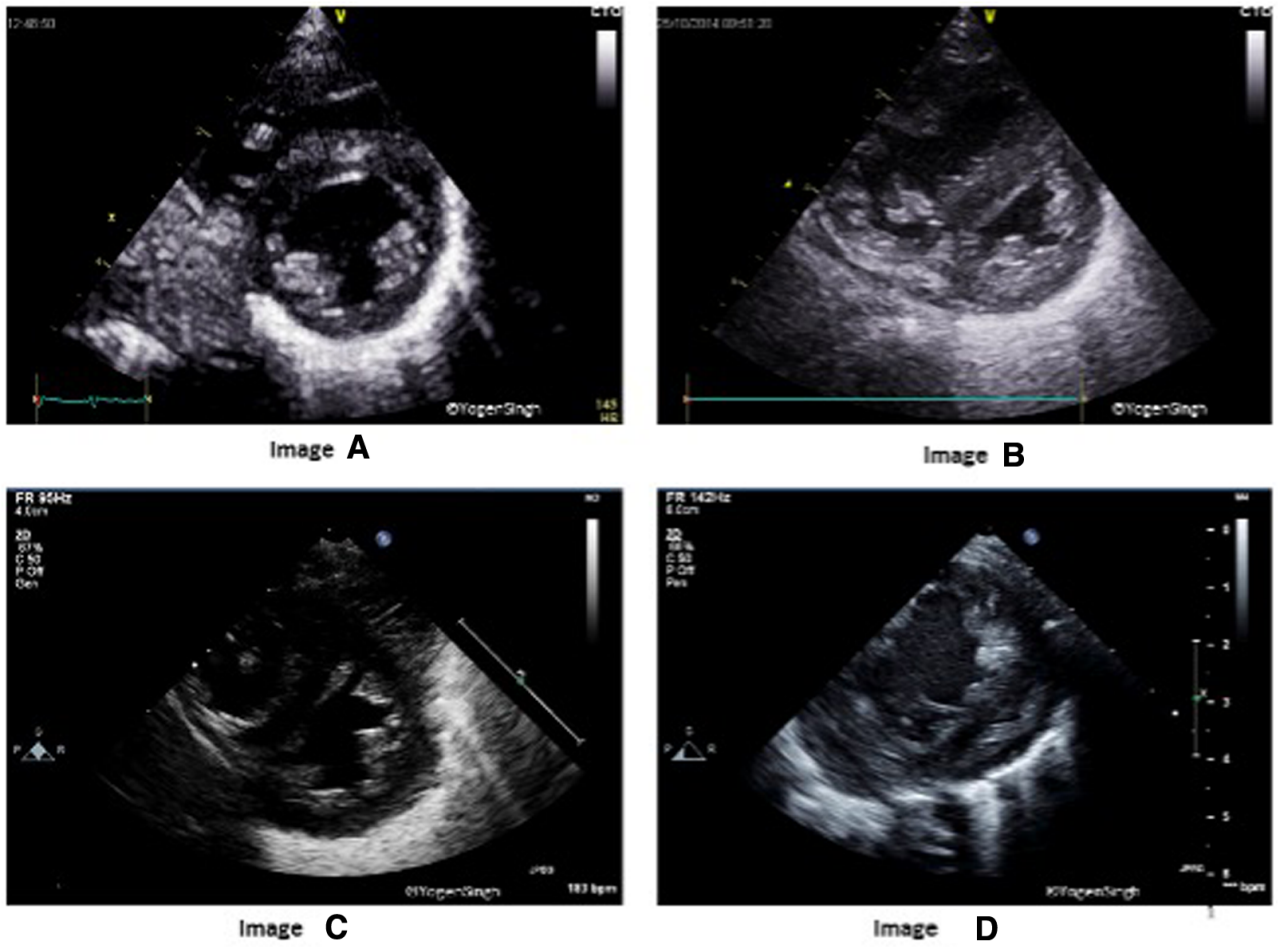
Interventricular septum (IVS) and left ventricle (LV) shapes in pulmonary hypertension. Image “**A**” shows normal IVS and LV shape with LV being a circular shaped structure on sweep-PSAX view. Images “**B**”, “**C**” and “**D**” show change in shapes with increasing flattening of IVS in presence of mild, moderate and severe pulmonary hypertension, respectively.

**Figure 3:**
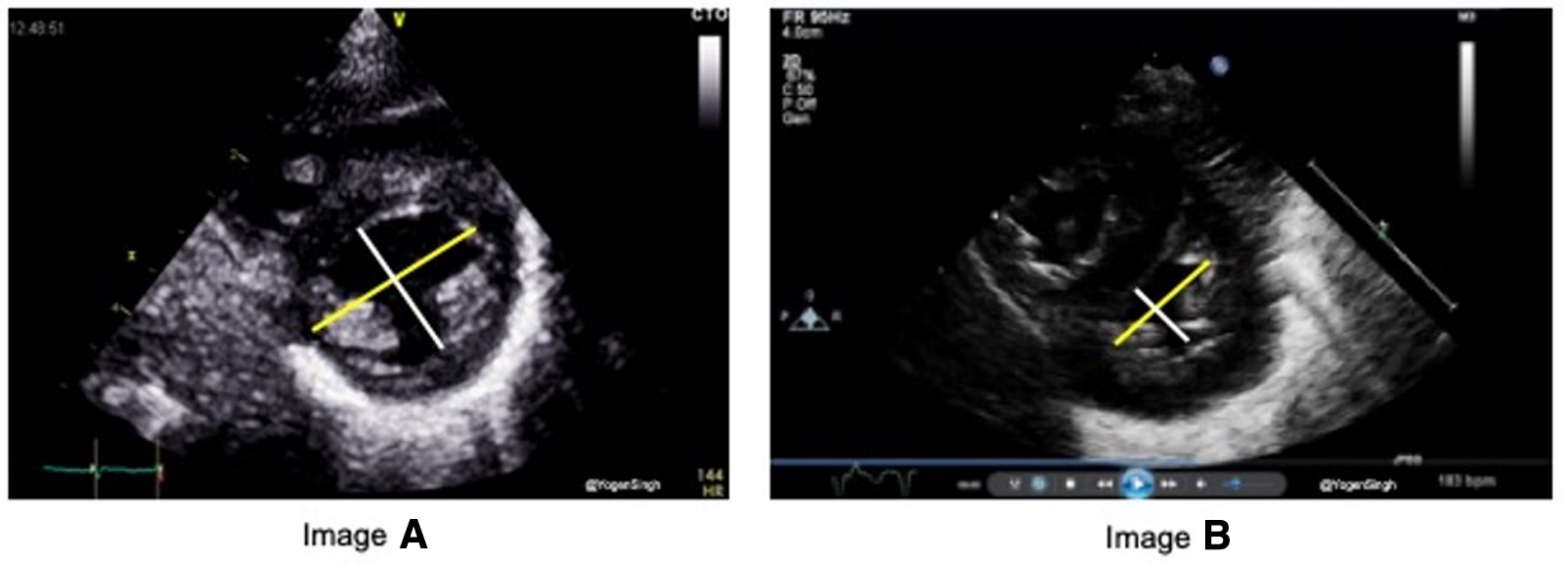
End-systolic eccentricity index (EI). EI is obtained by the ratio of left ventricle transverse (yellow line) and vertical (white) diameters at end-systole. Image “**A**” shows normal EI index and image “**B**” shows EI >1.3 in pulmonary hypertension.

Flattening of the interventricular septum (IVS) occurs when systolic pressure in the right ventricular pressure exceeds left ventricular pressure. A rounded IVS shape, defined as type I, indicates normal right ventricular pressures, whereas a flat or a bowed IVS at end-systole, defined as type II, and III respectively, are suggestive of right ventricular pressures between 50% and 100% or greater than 100% of the systemic systolic pressure, respectively (Figure 2) (10). Although accurate PASP cannot be estimated by studying IVS flattening, the presence of an abnormal septal position is one of the most adopted echocardiographic criteria for diagnosis of pulmonary hypertension in clinical practice, especially when there is no triscupid regurgitation (TR) (which is absent in up to 1/3 infants with PH) or patent ductus arteriosus (11–16). A quantitative estimation of septal flattening and of right ventricular remodelling is provided by the end-systolic LV-EI, calculated as the ratio between left ventricle transverse and perpendicular diameters at mid-ventricular level from a parasternal short-axis view (Figure 3).

#### 2.1.3. Left ventricular output (LVO), left ventricular ejection fraction (EF) and fractional shortening (FS)

Right ventricle (RV) hypertrophy and/or dilatation, as commonly seen in infants with pulmonary hypertension, will affect left ventricle (LV) filling and function because of inter-ventricular functional interdependence. Left ventricular EF, FS and LVO are routinely used parameters for the evaluation of the left ventricular performance, which are also altered in presence of RV dysfunction due to interventricular dependence.

LVO is the product of cross-sectional area of left ventricular outflow tract (LVOT), measured from the parasternal long-axis view at end-systole by measuring leading edge to leading distance at level of aortic annulus, LVOT velocity time integral (VTI) assessed by pulse-wave Doppler in apical five-chamber view and the heart rate. In the presence of ductal shunting with left to right shunt, LVO calculation is overestimated due to contamination from transductal shunt.

FS is measured from M-mode tracings or 2D imaging in parasternal long-axis view just distal to the tips of mitral valve leaflets, or in parasternal short-axis view at papillary muscles level, and is calculated as percentage ratio between the LV end-diastolic and end-systolic diameters. EF is calculated using biplane measurements of LV volume from apical four-chamber and two-chamber views, and is calculated as percentage ratio between end-diastolic and end-systolic volumes (17).

#### 2.1.4. RV to LV ratio

**Figure 4:**
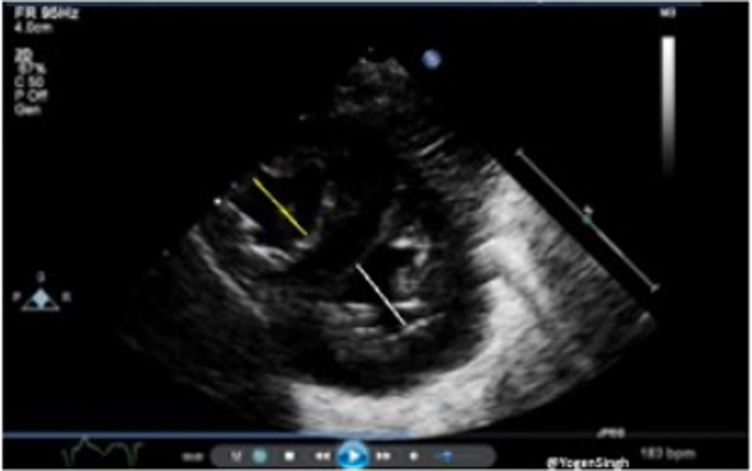
Right ventricle (RV) to left ventricle (LV) end-systolic ratio measurement in the parasternal short-axis view.

Normally, LV size is bigger than RV in children and adults. RV/LV ratio of more than 1 suggests PH, and it is best calculated in the parasternal short-axis view on 2D mode (Figure 4) (18).

#### 2.1.5. Right atrium and ventricular size, and RV fractional area change

In presence of PH, hypertrophy and/or dilatation of RV and right atrium is common and this could be measured using echocardiography (18). RV function can be quickly assessed from its size, wall thickness, and contractility by “eyeballing” (qualitative assessment). The shape of the RV makes RV function assessment more challenging, even in the expert hands. Fractional area change (FAC) can be calculated using echocardiography in RV-focused apical 4-chamber view or in a sub-costal modified parasternal short axis views aimed at visualising all three parts of RV (inflow, body and outflow). More commonly, in clinical practice RV function is objectively assessed by measuring tricuspid annular plane systolic excursion and/or tissue Doppler imaging as described below.

#### 2.1.6. Tricuspid annular plane systolic excursion (TAPSE)

**Figure 5:**
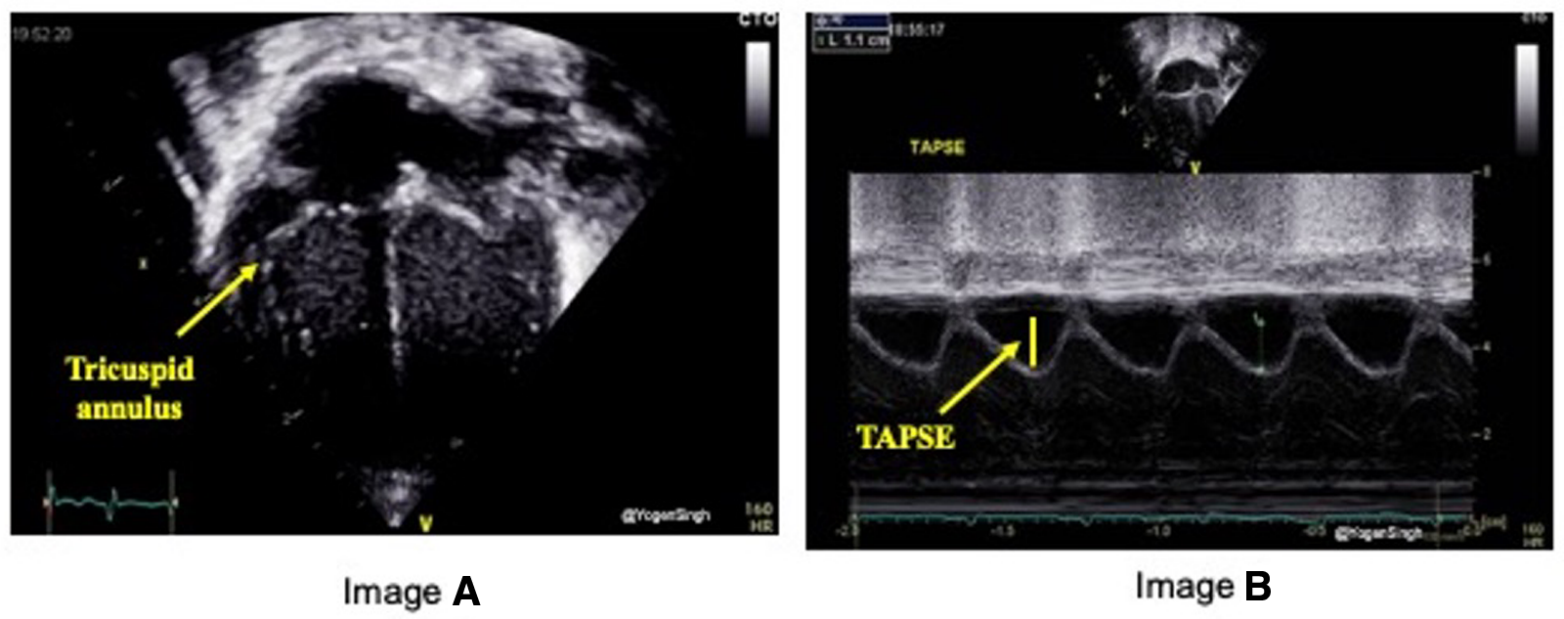
Tricuspid annular plane systolic excursion (TAPSE) measurement on echocardiography. TAPSE reflects the longitudinal movement of the tricuspid annulus towards apex in systole measured on M-mode (image “**B**”) in Apical 4-chamber view (image “**A**”).

TAPSE is defined as the excursion of the tricuspid annulus from its highest position to the systolic peak descent toward the apex and provides an estimate of RV contractility. It is measured using M-mode echocardiography in the apical four-chamber view (Figure 5). TAPSE evaluation has been shown to reflect RV systolic function well. It's easy to measure on bedside and reproducible even in in less cooperative patients with suboptimal ultrasonographic windows, making its use particularly suitable in the neonatal population.

#### 2.1.7. Tissue Doppler imaging (TDI)

TDI applies Doppler to measure movement of the myocardium, which follows a directional contraction and relaxation pattern, and to evaluate the timing of myocardial events in the cardiac cycle. TDI can be evaluated from apical 4-chamber view, placing the ultrasound beam along the left lateral, septal and right lateral walls.

**Figure 6:**
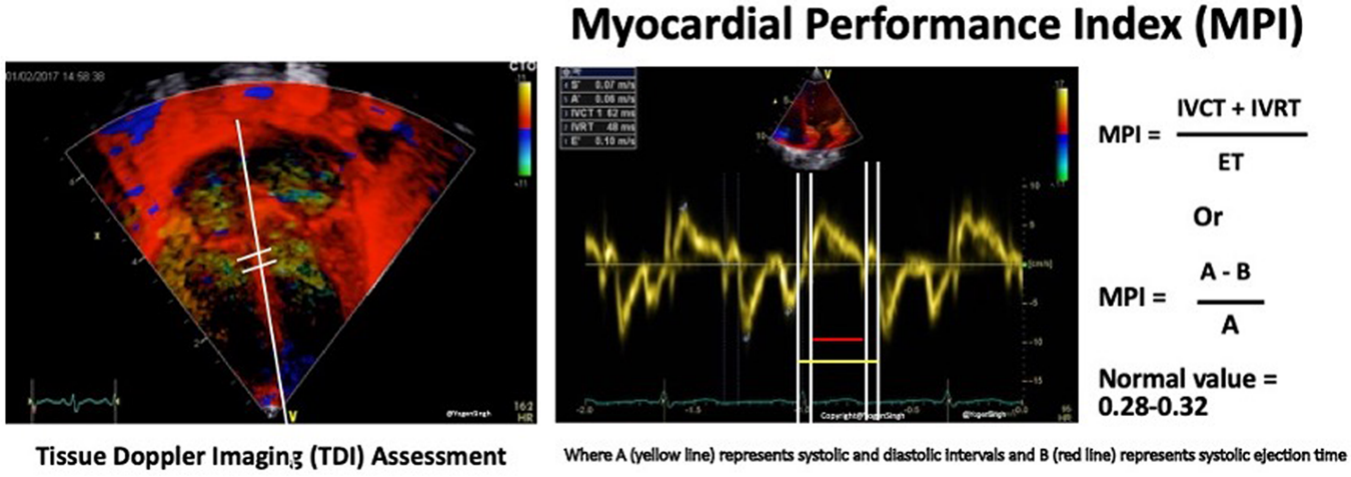
Tissue Doppler imaging (TDI, *left*) assessment of myocardial performance index (MPI, *right*).

The most frequently used time index by TDI is the myocardial performance index (MPI, Figure 6), which is assessed from the left lateral and right lateral hinge of the atrio-ventricular plane and derived according to the following formula:

$$\mathrm{MPI}=\frac{\mathrm{time}\;\mathrm{interval}\;(\mathrm{isovolumic}\;\mathrm{contraction}+\mathrm{isovolumic}\;\mathrm{relaxation})}{\mathrm{time}\;\mathrm{interval}\;(\mathrm{ejection}\;\mathrm{phase})}$$

Other TDI measurements include peak systolic excursion velocity (*s*’), early diastolic velocity (*e*′), and late diastolic velocity during atrial systole (*a*′). The ratio between the inflow velocity through tricuspid or mitral valve *E* and *e*′ velocity (*E*/*e*′) assesses the ventricular filling pressure, which is increased in case of diastolic ventricular dysfunction (19).

#### 2.1.8. Pulmonary artery acceleration time (PAAT) and PAAT to RV ejection time (PAAT:ET) ratio

**Figure 7:**
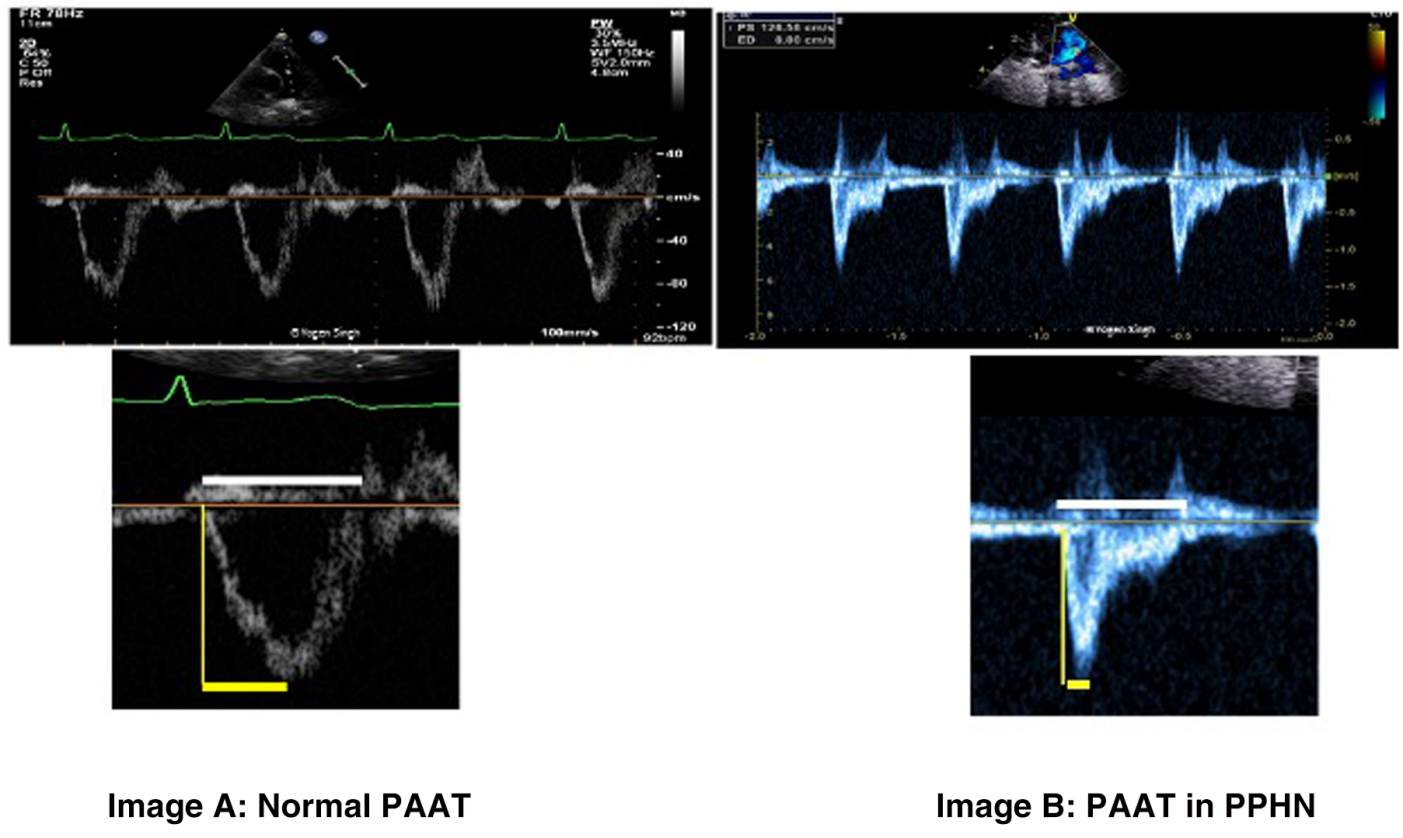
Pulmonary artery acceleration time (PAAT) measurement. Image “**A**” shows normal ratio of PAAT and right ventricular ejection time (RVET) in a normal child. Image “**B**” shows significantly decreased PAAT and increased PAAT/RVET ratio suggestive of pulmonary hypertension. “Dicrotic notch” may be seen on pulse-wave Doppler spectral in the pulmonary artery.

PAAT is defined as the time interval between systolic pulmonary arterial ejection and peak flow velocity (Figure 7) and can be assessed using pulse-wave Doppler in main pulmonary artery. It can be used as independent parameter or as a ratio with RV ejection time (PAAT:RVET).

#### 2.1.9. Speckle tracking

**Figure 8:**
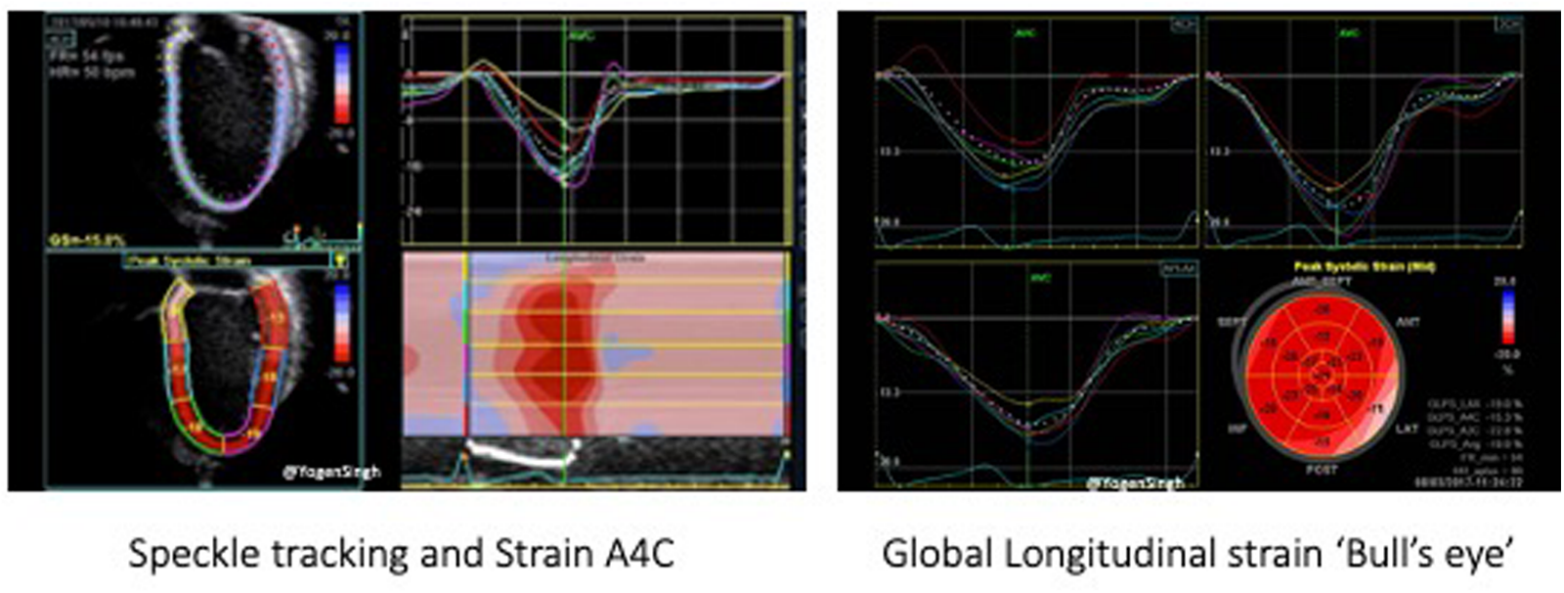
Myocardial strain measurements on two-dimensional speckle tracking echocardiography.

Two-dimensional speckle tracking echocardiography (STE) tracks speckles in the 2D plane to measure the myocardial shape changes in any direction within such plane between end-diastole and end-systole. Myocardial strain is a dimensionless index defined as the relative myocardial deformation and is expressed as a percentage (Figure 8). The strain rate (SR) is the rate at which myocardial deformation occurs (20). SR is a relatively preload independent measure of function and, as such, it is unlikely influenced by left to right shunting.

### 2.2. Lung ultrasound parameters

#### 2.2.1. Lung ultrasound score (LUS)

From an alternative diagnostic tool to chest x-ray (21, 22), use of lung ultrasound has recently been developed into the so-called “functional” method (23), which uses semi-quantitative lung ultrasound scores (LUS). Lung ultrasonography in neonatal settings derives from the application of LUS scores previously described in adult population (24), subsequently modified and adapted to the characteristics of neonatal population, where these scores were initially studied to evaluate their predictive value in assessing need for exogenous surfactant in infants with respiratory distress syndrome (RDS) (25–30).

**Figure 9:**
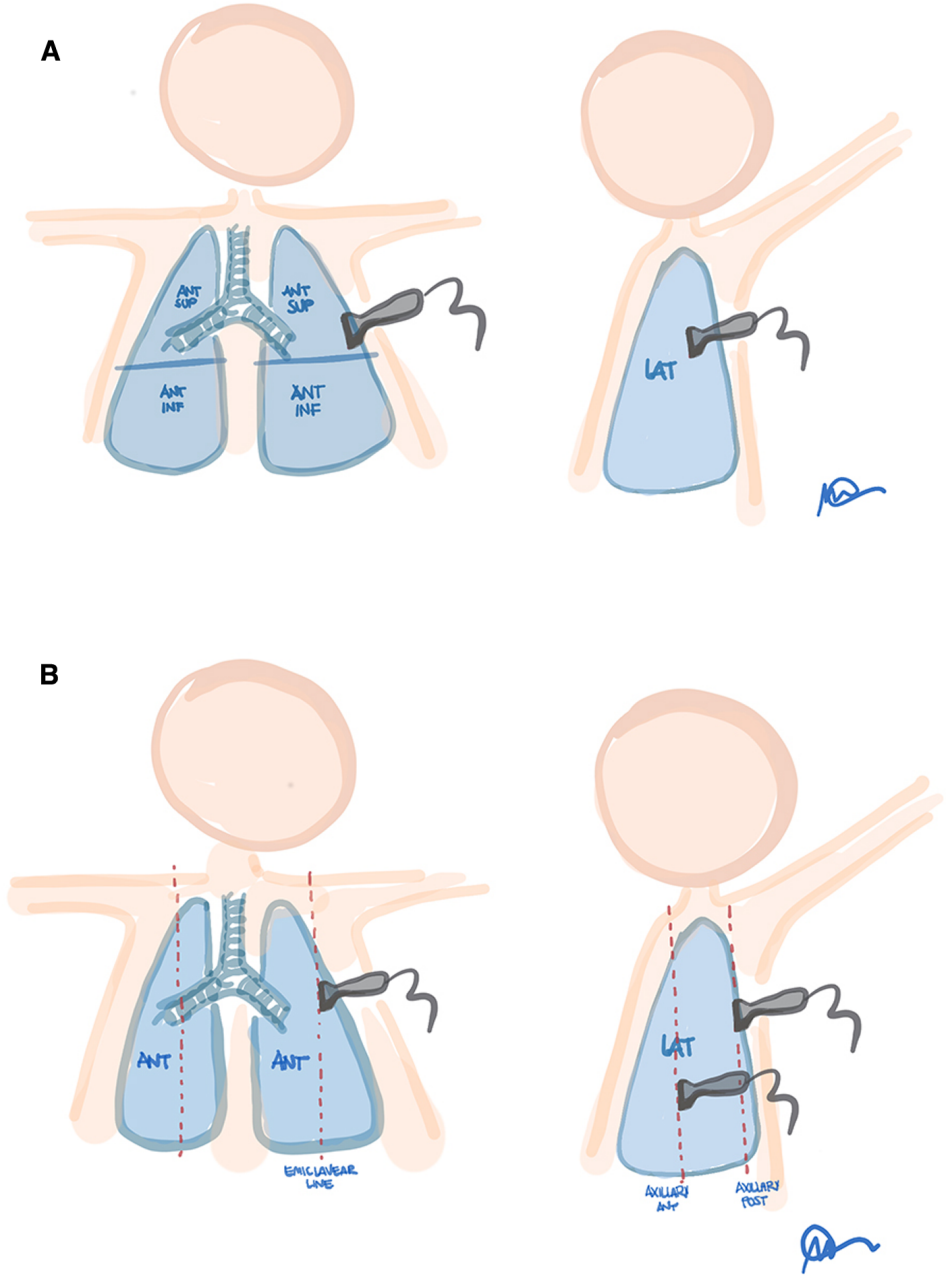
Proposed lung fields for 6-areas ultrasound scores. (**A**) Brat et al. (zones: antero-superior, antero- inferior and lateral for each hemithorax). (**B**) Raimondi et al. (zones: midclavicular, anterior axillary and posterior axillary lines, for each hemithorax). Modified with permission from Corsini et al. (32).

Lung ultrasound findings in neonatal RDS include pleural line thickness, reverberation artifact from interstitial fluid or decreased aeration, and parenchymal consolidation. To systemically evaluate the whole lung, chest is divided into several zones. Based on the type of ultrasound findings identified, a score is assigned to each area, whose sum provides an overall score, known as LUS (31). The most used scores are those proposed by Brat et al. (25) and Raimondi et al. (28), both based on a division of the chest into 6 zones as shown in Figure 9.

**Figure 10:**
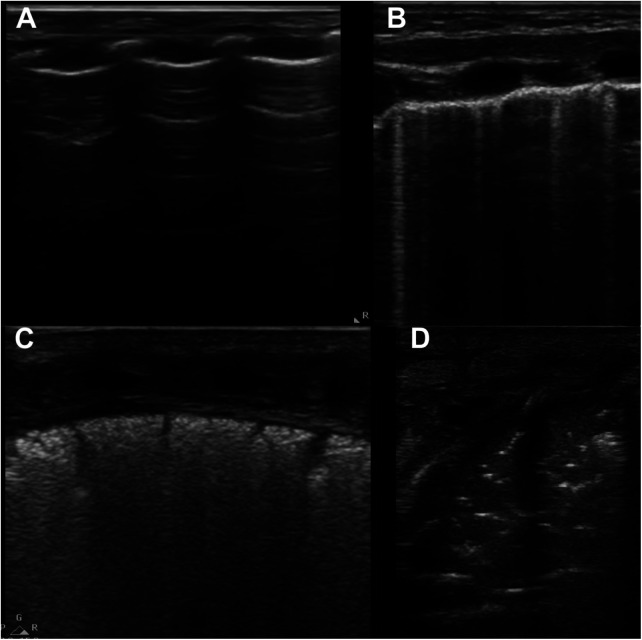
Lung ultrasound patterns used to calculate lung ultrasound scores. (**A**) score 0, A-pattern (defined by the presence of only A-lines); (**B**) score 1, B-pattern (defined as the presence of ≥3 B-lines); (**C**) score 2, severe B-pattern (defined as the presence of crowded and coalescent B lines with or without consolidations limited to subpleural space); (**D**) score 3, extended consolidation. Modified with permission from Corsini et al. (31).

The score attributed to each lung area is based upon the degree of aeration of lung parenchyma and it is evaluated semi-quantitatively. The commonly used method in clinical practice was the one proposed by Brat et al. (25) as illustrated in Figure 10. The score is attributed based on the following criteria: score 0: A-pattern (defined by presence of only A-lines; Figure 10A); score 1: B-pattern (defined as presence of ≥3 B-lines; Figure 10B); score 2: severe B-pattern (defined as presence of crowded and coalescent B lines with or without consolidations limited to subpleural space, Figure 10C); and score 3: extended consolidation (Figure 10D). Therefore, overall score can range between 0 (completely normal) and 18 (bilateral white lung findings with diffuse consolidations). Additional scoring systems have been further proposed in neonatal setting—the main differences are related to the zones in which chest is divided, both in terms of position and number.

## 3. Results

**Figure 11:**
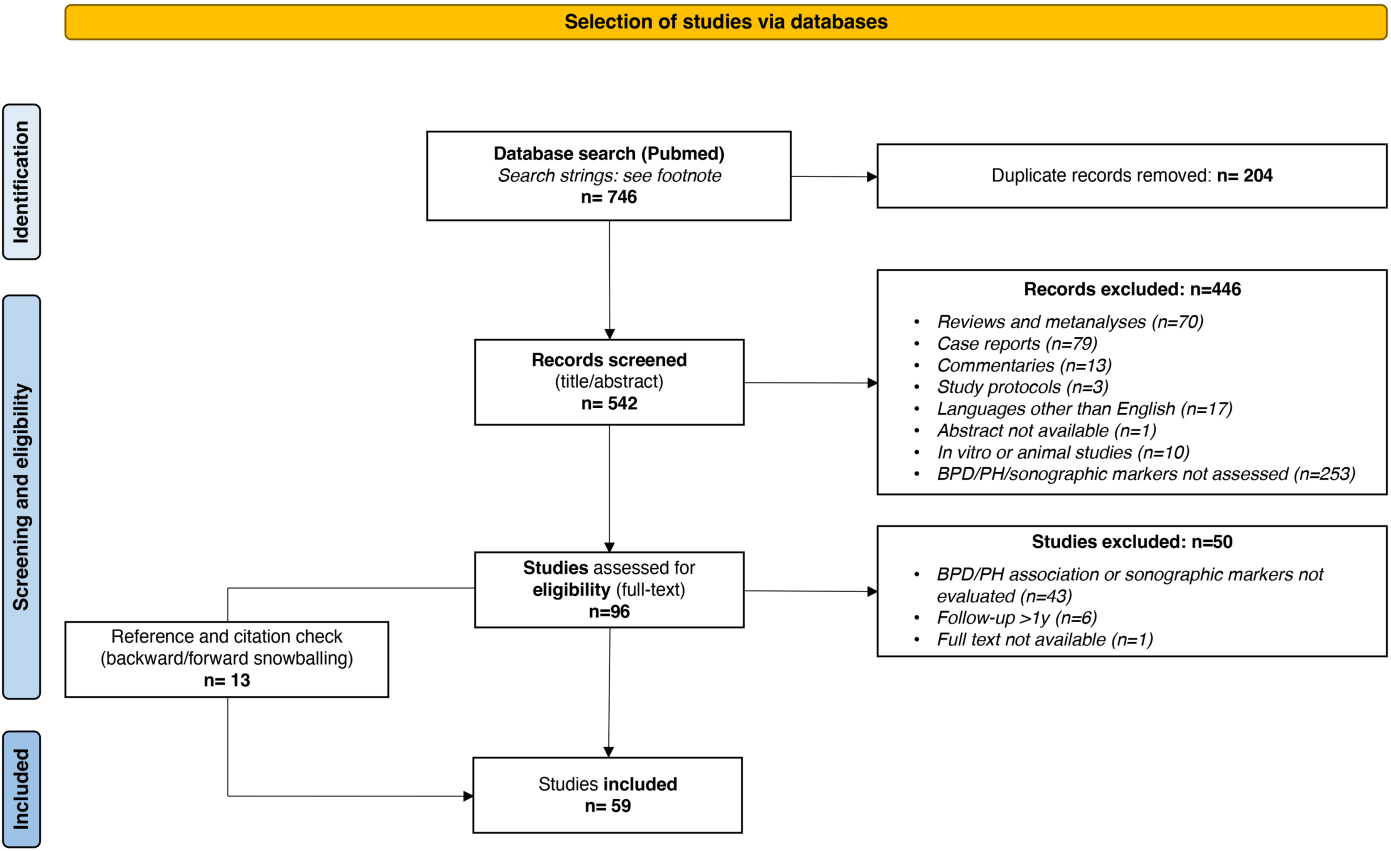
PRISMA flow diagram detailing the search and selection process applied for literature overview.

As shown in the PRISMA flow diagram (Figure 11), a total of 59 studies were included in this scoping review and categorized in the following sections: echocardiographic parameters for BPD association or prediction; echocardiographic parameters for PH association or prediction; lung ultrasound scores for BPD association or prediction.

The characteristics of the studies included are detailed in Supplementary Tables S1–S3, with particular reference to population characteristics, echocardiographic and lung ultrasound parameters assessed in each study, outcome definitions and results.

### 3.1. Echocardiographic markers for BPD association or prediction

Many studies have evaluated various echocardiographic parameters associated with BPD and its severity. The main relevant findings are summarized below, whereas the study details including the adopted BPD definitions are available in Supplementary Table S1.

Effort has been made to obtain these echocardiographic measurements early in first couple weeks after birth to predict late development of BPD at 28 days of life or 36 weeks postmenstrual age (PMA); however, the results are equivocal. We discuss the echocardiographic parameters that have been published in association with BPD.

• *TJRV and RVSP*. Mourani et al. reported a non-significant trend towards increased TRJV in association with increasing BPD severity (4%, 8.1% and 10.8% for mild, moderate and severe BPD, respectively) (13), while Choi et al. failed to demonstrate the correlation between TRJV, BPD development and its severity (33).
• *Septal flattening and LV-EI*. Current literature is concordant in describing a significant correlation between septal flattening and BPD severity. Mourani et al. had first observed a higher incidence of a flat or bowed IVS at 7 days in infants who subsequently developed severe BPD at 36 weeks PMA, although this finding did not reach statistical significance (13). Then a large study has shown that a flattened or bowed IVS (type II or III) at 7 days of life in extremely preterm neonates has proved to be an independent predictor of moderate to severe BPD at 36 weeks PMA with a sensitivity and specificity of 62.5% and 85%, respectively (34). Moreover, in infants with severe BPD with a PMA between 34 and 44 weeks, a flattened or bowed IVS was associated with an adjusted 1.9-fold increase in mortality rates (35). One should be aware that PVD seen in infants with BPD is not the only cause of abnormal septal position; in addition, volume overload from intracardiac shunting may lead to a flattened septum. As an example, an abnormal septal position after term PMA has shown a mild sensitivity (85.7%) and a poor specificity (25%) in correlation with high pulmonary vascular pressure in preterm infants with BPD when compared to cardiac catheterization; however, after the exclusion of infants with patent ductus arteriosus (PDA) and ventricular septum defects, the sensitivity and specificity of septal flattening increased to 91.7% and 100%, respectively (36). With regard to LV-EI, no difference between BPD infants without PH and preterm controls was found at a median PMA between 38.5 and 45 weeks (37). However, in the case-control study by Ehrmann et al. evaluating multiple echocardiographic parameters of right ventricular mechanics in relation to BPD severity, LV-EI was found to be the only objective parameter that was independently associated with BPD severity (severe vs. no BPD: OR 1.8) at 36 weeks PMA (12). A significant association between the development of severe BPD and LV-EI has also been reported by Seo and Choi in a small cohort of preterm infants over 36 weeks' PMA or before hospital discharge (38).
• *LV EF, FS and LVO*. While a significantly increased LV FS has been recently documented at near-term age in BPD infants compared to controls (39), no difference in EF values was reported by Akcan et al. in preterm-born infants with and without BPD aged 6–12 months, nor between varying degrees of BPD severity (40). A significantly higher LVO, indexed for the body weight, has been previously reported in preterm infants with BPD on day of life 28 (39). A prospective study has found that higher LVO during the first 2 weeks of life may predict BPD development later in life (41). Nevertheless, whether this association was indirectly mediated by the effects of a hemodynamically significant PDA, whose prevalence was significantly higher in infants developing BPD, was not clarified.
• *Right atrial and ventricular areas and RV-FAC*. Levy et al. investigated RV areas and RV-FAC from first 24 h after birth to 36 weeks' PMA in preterm infants with no or mild BPD vs. moderate to severe BPD (42). While no difference was observed between 2 groups over first 3 days of life in RV end-diastolic and end-systolic areas, but by 32 weeks PMA both areas significantly increased amongst infants with moderate or severe BPD. Similarly, no difference in RV-FAC between these study groups was found on day 1 and 3, whereas by 32 weeks PMA RV-FAC significantly decreased in infants with moderate and severe BPD compared to the control cohort. These differences were maintained at 36 weeks PMA, when BPD was diagnosed. Similar findings at 36 weeks were reported by Sehgal et al. (43), whereas other studies failed to confirm this association (12, 39). With regard to BPD severity, Haque et al. reported no significant difference among infants with none/mild, moderate and severe BPD at term PMA (44). At later follow-up assessments, significantly lower RV-FAC in ex-preterm infants with BPD compared to controls have been reported at 1 year CA (45, 46).
• *PW-MPI*. Higher RV PW-MPI have been reported at 7, 21 and 28 days of life in infants who developed BPD compared to those who did not (47). Similarly, higher PW-MPI values were observed in neonates with severe BPD compared to those with a mild disease or no BPD at (48) or after 28 days (38), but not during the first 2 weeks of life (38). The latter study also reported a significantly higher proportion of infants with a PW-MPI >0.38 in association with severe compared to mild BPD after 28 days of life (38). When assessed in a multivariate analysis, RV PW-MPI on day 7 (49) and day 28 (48) was found to act as an independent predictor of BPD or death.
• *TDI*. With regard to LV TDI velocities, a lower *E*′-wave velocity was observed in infants with mild BPD compared to no BPD at both 1 and 28 days of life (50). As for the RV, significantly higher values of RV TDI-MPI at 36 weeks PMA (43) were observed in preterm infants who developed BPD compared to those who did not. A significant, positive correlation between RV TDI-MPI, septal TDI-MPI and increasing BPD severity has also been reported (33). On the contrary, other studies failed to demonstrate a significant association between RV TDI-MPI and BPD severity at ≥36 weeks' PMA (12, 51) or earlier (48). Beside high RV TDI-MPI, a significantly higher RV *E*/*e*′ ratio and reduced tricuspid *e*′, *a*′ and *s*’ velocities have been reported at 28 days of life and in near term infants with BPD compared to controls (39). An increased *E*/*e*′ ratio has also been reported at ≥36 weeks' PMA in neonates with severe BPD compared to those with mild or no BPD (43, 52), with increasing values at increasing BPD severity (52). In a multivariate model including, among other functional echocardiographic parameters, lateral tricuspid *e*′ and *a*′, the latter velocity was found to be significantly lower in neonates with BPD compared to controls (51). Conversely, no difference in RV TDI velocities and ratios was found after 36 weeks' PMA (44) or at earlier phases (48) in other small cohorts of neonates with different grades of BPD severity.
• *TAPSE*. Using TAPSE for evaluating BPD risk and severity in preterm infants remains controversial. Attempt has been made to evaluate TAPSE as a BPD predictive tool: Neumann et al. reported a significantly lower TAPSE at 7 days of life in very preterm infants who died or developed BPD compared to controls (49). Sehgal et al (43) first described a significantly lower TAPSE at 36 weeks PMA in a small cohort of preterm neonates with BPD compared to controls. This finding was confirmed by Mendez-Abad et al. in a multivariate model including GA, PMA and RV TDI parameters (51). A significantly lower TAPSE in BPD infants compared with preterm controls has also been reported by Erickson et al. at 32 weeks PMA and at 1-year CA follow-up (46). Conversely, no difference in TAPSE values at 28 days of life and near term was observed between infants with and without BPD (39), and other studies failed to demonstrate a significant association between TAPSE values and increasing BPD severity (12, 38, 44).
• *PAAT and PAAT:ET ratio*. In 2017, Koestenberger et al. have provided normal reference values and *z*-scores for PAAT on a large pediatric cohort, including 113 neonates (53). Data in relation to BPD in preterm infants remains controversial. An association between PAAT:ET ratio and BPD was first described by Gill et al, who proposed a PAAT:ET ratio cutoff of 0.54 on day 7 (predictive value 78%) to predict BPD development (54). A few years later, Su et al. reported significantly lower PAAT:ET ratio from day 7 to 28 in BPD infants compared to controls, and reported a predictive value towards BPD of 82.8% for values <0.46 on day 7 (55). Significantly lower PAAT and PAAT:ET ratio on day 2, but not on day 1 or 5–7, have been reported in BPD infants compared to no BPD (32); however, in this study, receiver operating characteristic curve analysis failed at determining specific cutoff values of these parameters during the first week of life in predicting BPD. Significantly lower PAAT and PAAT:ET ratio in infants with BPD compared to controls have also been reported at 32 (32, 46), 36 (32) and 40 weeks PMA (56). Conversely, Aldana-Aguirre et al. reported significantly lower PAAT in BPD infants vs. no BPD at near-term age, but not on day 28, and no difference in PAAT:ET ratio at both assessments (39). Moreover, when PAAT and PAAT:ET ratio were assessed in relation to BPD severity, no significant difference among infants with different BPD severity was observed at 36 weeks PMA (12) or earlier (48). With regard to follow-up studies, significantly lower values of PAAT:ET at term PMA (57) and of PAAT and PAAT:ET ratio at 1 year CA (45, 46) have been described in former preterm infants with BPD compared with those without BPD.
• *Speckle tracking*. Significantly altered rotational mechanics of the LV were observed at 28 days in infants with BPD compared to controls; however, only the untwist rate persisted significantly lower in the BPD group (39). Breatnach et al observed an increased LV twist and torsion in BPD infants at 36 weeks PMA (58). BPD neonates also showed decreased circumferential early diastolic SR at 28 days and higher circumferential late diastolic SR at near-term follow-up, suggesting the presence of LV diastolic dysfunction compensated by an increased velocity in atrial contraction (39). Czernik et al. investigated LV STE throughout the first month of life in preterm infants as potential BPD predictor, observing significantly higher global longitudinal strain (GLS) and longitudinal systolic SR (GLSR) of the mid-left wall on day 1 and day 7 in VLBW infants who later developed BPD compared to those who did not (41). Lehmann et al. reported different significantly decreases in torsion from 32 to 36 weeks PMA, but not later, between infants with BPD/PH/PDA (with BPD being the most represented diagnosis) and uncomplicated controls (59). With regard to RV, Erickson et al. documented significantly lower values in magnitudes of free wall longitudinal strain (FWLS), FLWS rate and basal, mid-ventricular and apical longitudinal strains at 32 weeks PMA in infants who developed BPD compared to those who did not (46). Similarly, James et al. reported lower RV basal longitudinal strain and late diastolic strain rates, but not in LV or septal strain parameters, in BPD infants at 36 weeks PMA, which coincided with BPD diagnosis (60). At 1 year CA, former preterm infants with BPD had significantly lower RV free wall longitudinal strain (FWLS) compared to preterm controls infants, while no difference was observed in LV FWLS (45). This finding was further confirmed in a later study by Erickson et al, where lower magnitudes of RV segmental longitudinal strains and FLWS rate were also reported in the BPD group (46).

### 3.2. Echocardiographic markers for PH association or prediction

All the above described BPD-associated echocardiographic parameters are indicators for the presence of PVD and PH. It is a logical association as an abnormal development of pulmonary microvasculature is a main pathophysiological characteristic of the “new BPD”. As such, the development of PH is frequent, especially in the most severe BPD cases, and is burdened by significantly increased rates of morbidity and mortality (61). These echocardiographic parameters may suggest PH at the time of BPD diagnosis or increased BPD severity; however, having abnormal values early in life (at 7–14 days of life) may not consistently predict BPD development later (at ≥28 days of life). Although having PH early in life is more related to post-natal transition and it does not necessitate future development of BPD, some studies have reported a potential association between these conditions (62, 63). The correlation between early and late PH is also quite controversial. While some studies have identified early PH as a possible independent risk factor for subsequent development of late PH (13, 64), others failed to find a significant association (65, 66). However, having late PH at time of BPD diagnosis at 36 weeks PMA (or at 28 days of life) is associated with a persistent PH course for months and even years, requiring long-term surveillance (67). A prompt detection and appropriate treatment could improve the outcome in these neonates; for this reason, several echocardiographic markers have been assessed to diagnose or predict PH development in BPD infants. The main PH relevant findings are summarized below, whereas the study details including the adopted PH definitions and the related timing of diagnosis, are provided in Supplementary Table S2.

• *TRJV and RVSP*. A TRJV >2.8 m/s is equivalent to a RVSP of 36 mmHg and is often considered as part of PH definition (61). Nonetheless, the presence of TRJV during early neonatal period does not necessarily predict PH development, while it has been reported that TRJV can be measured only in a variable proportion of infants with BPD and suspected PH (68). Hence, by itself, a TRJV >2.8 m/s has not shown to be a reliable PH predictor in BPD infants. In a large, retrospective multicentric study selectively evaluating preterm infants with severe BPD, a RVSP >40 mmHg between 34 and 44 weeks' PMA was associated with a 2.2-fold increase in mortality, even after the adjustment for multiple covariates (35).
• *LV-EI*. A LV-EI ≥1.15 was found to accurately correlate with the presence of PH, defined by the echocardiographic presence of qualitative septal ﬂattening, RVSP >36 mmHg or RVSP/systemic systolic blood pressure >0.5 in extremely preterm infants with BPD at term evaluation (14). Compared to BPD without PH and no BPD, BPD-PH was associated with a higher LV-EI at >36 weeks (37); in particular, consistently with the previously mentioned threshold of 1.15, systolic and diastolic LV-EI values in the BPD-PH group were 1.46 and 1.47 respectively, whereas the control group had a LV-EI. Moreover, in a small cohort study comparing LV-EI between BPD infants with or without PH, the former showed values remarkably ≥1.15 at both 3 and 12 months of age (69).
• *RV/LV ratio*. In a cardiac catheterization study on post-term PH infants without a PDA, a significant, positive correlation between this ratio and PVR was observed (36). In the study by Sallmon et al., infants with PH showed significantly higher RV/LV ratio at 3 and 12 months compared to those without PH (69). Conversely, Savoia et al. observed no difference between PH and no PH cohorts at both 36 weeks PMA and at 6 months CA follow-up (70).
• *Right atrial and ventricular areas and RV FAC*. A dilated area of the right atrium and increased end-diastolic and end-systolic RV areas were observed at 36 weeks' PMA in BPD infants with signs of PH compared to those with no PH (16). Similar findings of enlarged right sections, along with a significantly reduced RV FAC at 36 weeks PMA have been recently reported in infants who developed signs of PH at hospital discharge (11). At around 1 year CA, lower RV FAC was observed in infants with late PH compared to controls (16, 45).
• *TDI*. Data evaluating the association between TDI parameters and PH are limited. Savoia et al. found no difference in several TDI parameters (namely, LV lateral wall *E*, *S*’, mitral *E*/*E*′, tricuspidal *E*, *A*, *E*/*A* and *E*/*E*′, RV free wall *E*′ and *S*’) between BPD infants with and without PH at both 36 weeks PMA and 6 postnatal months (70). No difference was also observed by Behere et al. at term age (16), whereas Richardson et al. reported lower mitral, basal IVS S' and tricuspidal *S*’ in PH preterm infants compared to controls scanned at a median age of 66 postnatal days (71).
• *TAPSE*. a significantly higher prevalence of TAPSE <5 mm within the first 2 weeks after birth was documented by Seo and Choi in preterm neonates with BPD developing PH compared to those who did not (38). At later evaluations, Richardson et al. described lower TAPSE at a median age of 66 days in PH preterm infants compared to matched controls (71) whereas Sallmon et al. reported significantly lower TAPSE in PH infants compared to no PH at 3 and 12 months (69).
• *PAAT and PAAT:ET ratio*. A borderline negative correlation between PAAT and PVR, assessed by cardiac catheterization after term-equivalent age, has been observed in the absence of a PDA (36). In their validation study on normal PAAT values in the pediatric population, Koestenberger et al. evaluated PAAT in a small PH cohort including patients aged 1 month to 18 years, and reported that a PAAT value <−2 age-specific z-score proves a good sensitivity and specificity to detect PH (53). While no difference in PAAT and PAAT:ET ratio during the first week of life was observed between infants with and without PH, all the PH infants at 32 and/or 36 weeks PMA had decreased PAAT and PAAT:ET ratio at both time points when compared to those without PH, even after adjustment for the presence of BPD, and a PAAT <47 msec and PAAT:ET ratio <0.28 at 32 weeks PMA were identified as cut-off values for PH detection at 36 weeks PMA (32). In a recent follow-up study on BPD infants with and without PH, both PAAT and PAAT:ET ratio were found to be significantly lower at both 36 weeks PMA and 6 months CA in the PH group (70). In a small PH cohort, PAAT was significantly lower in BPD infants with PH at 3 and 12 months compared to no PH (69). At 1 year follow-up, significantly reduced PAAT and PAAT:ET ratio values have been further reported in former preterm infants with echocardiographic evidence of PH compared to controls with no PH (32, 45).
• *Speckle tracking*. In a large STE study including 1-year follow-up, infants with BPD and/or PH showed a persistently lower RV and septal GLS up to 12 months PMA compared to healthy preterm controls, while LV GLS and GLSR did not differ (15). Similar findings were documented at 6 months PMA in preterm-born infants with BPD and PH compared to those without PH (72), thus suggesting that the presence of PH leads to a persistent impairment of RV systolic function.

### 3.3. Lung ultrasound markers for BPD association or prediction

Most of the available literature on lung ultrasonography assessments for BPD association or prediction is based on different LUS, whereas only a few studies have investigated other sonographic findings, which are discussed in the following paragraphs.

• *LUS*. Although some authors report that the use of LUS does not add to prediction of BPD development compared to models based on GA (73), a growing body of literature, detailed in Supplementary Table S3, is consistent in reporting the potential role of lung ultrasound, performed at different timing between the first 3 days of life up to 28 days in prediction of late BPD at 36 weeks PMA (28, 74–83). The predictivity of LUS towards development of BPD was also confirmed in a recent meta-analysis (84). From the analysis of seven studies on over 1,000 neonates, LUS showed good diagnostic accuracy in predicting BPD at 7 and 14 days of life (area under the curve, 0.85–0.87; pooled sensitivity, 70%–80%; pooled specificity, 80%–87%) (84).
  The number of chest zones to be evaluated are not standardized. Loi at al were the first investigators to compare BPD prediction using classical (anterolateral fields) vs. extended LUS (anterior, lateral and posterior fields), and they reported that both LUS and extended LUS have the same ability to predict the development of BPD and their best performance is at 7 days (82) (see Supplementary Table S3). The authors also proposed a combined model where both LUS and GA were taken into account by calculating GA-adjusted LUS (ratio between LUS and GA), however, no significant difference between GA-adjusted extended and normal LUS was observed. Recently, Sun et al. evaluated the association between BPD severity and a modified Brat score which also included retro-hepatic and retro-splenic views obtained from subcostal scans. Although the modified score appeared to better correlate with BPD severity at 36 weeks than the classic one, its predictive capacity at 7–14 days of life was not explored (85). The two different scoring types (6 vs. 10 chest fields) have also been evaluated in a meta-analysis (84) which recommended using simpler one, since no significant between-scores difference was detected in terms of BPD prediction.
  Other recent studies have explored the association between LUS and BPD; however, in these studies, reference cut-offs and sensitivity and specificity parameters for the LUS used have not been provided (86–88).
• *Other findings*. A small number of studies, most of which dating back to the 90s and early 2000s when the use of LUS had not developed yet (89, 90), evaluated the association between BPD development and the evidence of specific findings at lung ultrasonography. Avni et al. described the persistence of retro-diaphragmatic hyperechogenicity at 28 days of life in all the infants who later evolved to BPD, whereas this finding was absent in 95% of neonates who did not developed BPD (89). Similar findings were later observed by Pieper et al., who also identified day 9 as the day of assessment with highest predictor values towards BPD development (90). More recently, Gao et al. recently reported a significant association between BPD development and the presence of a section of pulmonary consolidation with subpleural structure disorders, fragment-like strong echoes, and irregular weak echo areas at lung ultrasound performed weekly from the first 72 h of life to 36 weeks PMA, which coincided with the timing of BPD diagnosis (91). Finally, Sun et al. reported a thicker pleura and a higher proportion of rough pleural lines, retrodiaphragmatic hyperechogenicity, small cysts above the diaphragm and of rough diaphragm in infants with moderate-to-severe BPD compared than none-to-mild BPD at 36 weeks PMA (92).

## 4. Discussion

Over the past decades, a growing body of evidence on the “new” BPD has shed light on the tight interplay existing between altered alveolarization and the disruption of pulmonary vascularization, leading to PVD. Consequently, non-invasive ultrasound techniques have been increasingly adopted to investigate the associated cardiovascular and pulmonary changes, with the goal of identifying echocardiographic markers predictive of BPD development before the pulmonary and vascular remodelling characteristic of this condition develop and hence serve to improve long-term outcomes.

Among echocardiographic parameters associated with BPD, the presence of IVS flattening and TDI indicators of impaired RV function have proved a better association, whereas data on other RV parameters, such as TRJV, PAAT and TAPSE, are still controversial. On the contrary, based on this available evidence, conventional LV parameters cannot be considered as reliable echocardiographic parameters for prediction of BPD. With regard to speckle tracking, although promising, to date this technique mainly appears a research tool and the related findings should be taken cautiously and interpreted in association with other echocardiographic parameters and in relation to the clinical context.

When BPD severity was evaluated, the use of RV TDI parameters has yielded encouraging results compared to other echocardiographic biomarkers, which could be due to the fact that severe BPD is often associated with PH and RV TDI is a good echocardiographic parameter for assessing RV function. However, limited studies have been done to evaluate the association of BPD severity and PH. The PH found at 36 weeks PMA or later in infants with a BPD diagnosis may persist for months and years and increase long-term morbidities and mortality. It is recommended to have PH surveillance with echocardiography in all preterm infants at 36 weeks PMA, which can help to determine post NICU discharge long-term surveillance schedule and PH treatment.

When available literature on echocardiographic parameters is globally examined, important limitations are evident. First, the available data are usually obtained from small study cohorts, or retrospective studies from a single center, which may contribute to limit generalizability of the observed results. Moreover, there is a remarkable variability in the timing at which the echocardiographic assessments are performed in the different studies (i.e., from the first days of life to term-equivalent age), and this significantly hinders to assess the predictivity of such echocardiographic parameters towards BPD or BPD severity. In particular, it should be noted that several studies have investigated the association between echocardiographic markers and BPD not only before the diagnosis is made, but also beyond the age at which BPD was diagnosed. Therefore, ultrasonography or echocardiography findings obtained during later period cannot be used for BPD prediction, but rather for the association between these biomarkers of PH and BPD.

Secondly, and equally importantly, the adopted definitions for BPD, its severity or PH differ among the published literature, thus further contributing to limit the comparability of results. In particular, a consensus on the definition of PH before 3 months of age has not been established. Hence, although many studies converged on the evidence of IVS flattening and/or RVSP >50% to define PH, if follows that the variability in PH definition poses a relevant bias for the identification of reliable echocardiographic predictors of PH based on the available literature. Moreover, several echocardiographic parameters are used for PH diagnosis itself and, as such, cannot be appropriately considered as PH predictors.

Thirdly, even though echocardiography is an excellent non-invasive bedside screening tool for PH, it is an operator dependent tool and pulmonary artery systolic pressure is estimated from the Doppler flow velocity and a guessed right atrial pressure (8). Cardiac catheterization remains the gold standard for diagnosing PH because it truly measures the pulmonary pressure (8). However, cardiac catheterization in a sick, preterm infant can be associated with mortality and morbidities. Echocardiography is routinely used as a bedside screening and diagnostic tool for PH and its follow up to assess severity or response to treatment, and it is being used all over the world. It is still recommended to perform cardiac catheterization data measurement and vasoreactivity testing before starting PH-targeted therapy when possible (93). Nevertheless, in clinical practice, not all patients with PH have cardiac catheterization performed to diagnose PH. So, one of the major limitations of many studies reviewed in our scoping review is lacking confirmatory PH data from cardiac catheterization. In clinical practice, echocardiographic data for PH is commonly not validated against the limited cardiac catheterization in preterm infants. McCrary et al. has showed that a high inter- and intra-rater agreement for the diagnosing PH in at-risk preterm infants by using standardized echocardiography reading protocol (94). However, more validated echocardiography studies against cardiac catheterization data are needed to solely depend on echocardiography to diagnose PH and to target treatment.

While early life echocardiograph parameters may not predict BPD, early lung ultrasound has shown promising results in predicting late development of BPD. Using lung ultrasound as a predictor is limited by the non-standardized approach in calculating LUS, as diverse studies used different number of chest zones and different LUS cut off to predict BPD. There are insufficient data to recommend a particular score over another for BPD prediction. The best score is certainly the simplest one, as it allows a better reproducibility and reduces newborn handling. For this reason, the inclusion of the posterior quadrants in LUS is still debated. On one hand, being BPD an inhomogeneous disease involving the severity-dependent portions of the lung (95), the evaluation of the posterior portions seemed to improve the performance of the LUS. On the other hand, the need to turn the neonate may represent a trigger for destabilization, and it has been recently shown that including the posterior quadrants does not increase the predictive capacity of LUS (79, 84). The data of greatest interest are those reported by lung ultrasound studies focussed on findings in early postnatal life (i.e., 7–14 days of life). However, the obtained results are influenced by several factors, including the used BPD definition (96–98) and the type of score adopted, which leads to a wide range of thresholds to predict BPD. Hence, it is advisable that a well-defined diagnostic protocol is adopted within each NICU, using the same LUS at the same times, unifying the decision-making cut-offs.

## 5. Conclusions

Traditionally, echocardiography has been used for the diagnosis and monitoring of PH in neonates and children. Infants with moderate to severe BPD have a high risk of developing PH and with high risk of mortality; hence, recently emphasis has been placed on early diagnosis and prediction of BPD and PH. Lung ultrasound at day 7 after birth may be a better predictive of late development of BPD. Although many echocardiographic parameters for assessment of pulmonary hypertension associated with BPD and its severity, however there are not precise echocardiographic indicators during the first 2 weeks of life to predict PH or pulmonary vascular disease in infants with BPD at 28 days of life or 36 weeks PMA. This may benefit from further prospective studies targeted as early echocardiographic markers predictive of late development of PH at 36 weeks PMA. Echocardiographic parameters are important long-term PH surveillance tools in infants with BPD to monitor the progression of PH for months after NICU discharge. Further prospective ultrasonographic studies and/or newer imaging modalities are needed to better understand the physiologic and developmental dynamics between the heart and lungs in preterm infants with BPD.

### 5.1. Take home messages

1. Several echocardiographic parameters, especially assessing RV function, have shown to be associated with the presence of BPD. However, assessment of such echocardiographic parameters at 1–2 weeks of age may not effectively predict later BPD development.
2. Abnormal RV echocardiographic parameters reflect associated PVD and PH when present, consistently with the pathophysiology of the “new BPD” involving pulmonary vasculature.
3. Echocardiographic finding of PH in BPD infants at 36 weeks' post-conceptional age or at 28 days after birth increases risk of mortality and long-term PH. Hence, it is recommended to have routine PH surveillance in all at risk preterm infants at 36 weeks PMA, including an echocardiographic assessment.
4. LUS assessment at 1–2 weeks of age shows promising results for BPD prediction.
5. More studies on sonographic markers, especially on echocardiographic parameters, are needed for the validation of the currently proposed parameters and the timing of assessment.
