# Supplementary material for: A scoping review of echocardiographic and lung ultrasound biomarkers of bronchopulmonary dysplasia in preterm infants
Source: Front Pediatr. 2023 Feb 10;11:1067323. doi: 10.3389/fped.2023.1067323 (PMC9950276; doi:10.3389/fped.2023.1067323)
Supplement: Supplementary file 1 [file Datasheet1.pdf]

Supplemental Table 1. Details of clinical studies investigating echocardiographic (ECHO) parameters for bronchopulmonary dysplasia (BPD) assessment or prediction, listed in chronological order by year of publication.

Author	Study sample	BPD definition	ECHO timing	ECHO parameters	Main findings
Gill and Weindling. *Arch Dis Child* (1993)	54 very low birthweight infants (BPD, n=34; controls, n=20).	Supplemental O_2 at 36 weeks PMA with characteristic radiographic findings	Days 1, 2, 3, 7, 14, 21 and 28	Pulmonary artery acceleration time to right ventricular ejection time ratio, corrected for heart rate (PAAT:ETc)	• Significant fall in PAAT:ET ratio in the BPD group<br>• Predictive value of a PAAT:ET ratio <0.54 on day 7 towards BPD: 78% (sensitivity 73%, specificity 65%): on day 28: 97% (sensitivity 88%, specificity 95%).
Benatar et al. *Arch Dis Child Fetal Neonatal Ed* (1995)	42 preterm infants <34 weeks (BPD, n=30; controls, n=12).	Supplemental O_2 at 36 weeks PMA	Mean 40 weeks PMA	PAAT, PAAT:ET and PAAT:ETc ratio	• Significantly lower PAAT, PAAT:ET and PAAT:ETc ratio in BPD infants compared to controls
Su et al. *Arch Dis Child Fetal Neonatal Ed* (1997)	72 preterm infants <1500g (BPD, n=21; no BPD, n=51)	Supplemental O_2 at 28 days with characteristic radiographic findings	Days 1, 2, 3, 7, 14, 21 and 28	PAAT:ETc ratio	• Significantly lower PAAT:ETc ratio from day 7 to 28 in BPD vs.no BPD infants (day 7, p<0.001; days 14–28, p<0.0001).<br>• In BPD infants, PAAT:ETc ratio fell significantly from 0.47 (standard deviation, SD 0.11) on day 7 to 0.41 (0.07) on day 28 (p=0.01).<br>• Predictive value of a PAAT:ETc ratio <0.46 on day 7 towards BPD: 82.8%, (sensitivity 54.5%, specificity 94.1%).
Subedhar and Shaw. *Arch Dis Child Fetal Neonatal Ed* (2000)	98 preterm infants ≤34 weeks (BPD, n=54; controls, n=44).	Supplemental O_2 for at least 28 days and beyond 36 weeks PMA with characteristic radiographic findings	36 weeks PMA, 40 weeks PMA, 1–13 weeks corrected age (CA), 14–26 weeks CA, 27–39 weeks CA, 40–52 weeks CA	PAAT:ET ratio	• Significantly lower PAAT:ET ratio from term to 39 weeks CA<br>• Negative association between BPD and PAAT:ET ratio (b=-0.0392, p<0.0001) confirmed after adjustment for gestational and postnatal age.

Yates et al. *J Ped* (2008)	21 preterm neonates (no/mild BPD, n=6; moderate BPD, n=7; severe BPD, n=8)	Supplemental O_2 at 36 weeks' PMA in infants ≥ 28 days<br><br>Mild BPD: no supplemental O_2; moderate BPD: FiO_2<0.3; severe BPD: FiO_2 ≥ 0.3 or PPV *(NICHD 2001)*	Mean 44.9 ± 19.1 weeks	• RV and LV tissue Doppler imaging (TDI) parameters and myocardial performance index (TDI-MPI)	• RV E/E' ratio: higher in severe (p=0.004) and moderate BPD (p=0.03) vs. no/mild BPD<br>• Correlation between BPD severity, RV E/E'(p=0.007, R^2 = 0.33) and LV-MPI (p=0.02, R^2 = 0.28)
Czernik et al. *PLoS One* (2012)	121 preterm infants (BPD, n=36)	Supplemental O_2 at 36 weeks' PMA	Days 2, 7, 14 and 28	• RV pulsed wave (PW)-MPI	• Higher PW-MPI in BPD compared to no BPD on day 7 (0.31[IQR 0.22–0.39] vs. 0.35[0.29–0.48], p=0.014), day 14 (0.23[0.17–0.30] vs. 0.35[0.25–0.43], p<0.001) and day 28 (0.21[0.15–0.28] vs. 0.31 [0.21–0.35], p=0.015).
Akcan et al. *Indian J Ped* (2013)	28 preterm neonates <32 weeks	NICHD 2001	6-12 (mean 9 ± 2) months	• Ejection fraction (EF) and fractional shortening (FS)<br>• LV end-diastolic (LVEDD) and end-systolic areas (LVESD)<br>• End-diastolic and end-systolic interventricular septum (IVS) thickness<br>• End-diastolic and end-systolic LV posterior wall thickness (LVPW)<br>• LV and RV TDI parameters and TDI-MPI	• TME': significantly lower in BPD infants<br>• TE/TME' and IVRT: significantly higher in BPD infants<br>• Significant correlation between TE, TE/TLE', TE/RVLE' and TE/TME' and BPD severity<br>• No difference in the other parameters
Czernik et al. *Plos One* (2014)	119 preterm infants <1500 g birth weight (BPD n= 36)	Supplemental O_2 at 36 weeks PMA	Days 1, 7, 14, and 28	• LV FS<br>• Left ventricular output (LVO)<br>• Speckle tracking parameters (global longitudinal systolic strain rate [GLSSR], longitudinal systolic strain rate [LSSR])	• GLSSR and LSSR for the mid left wall: significantly higher on day 1 and 7 in BPD infants<br>• LVO: significantly higher on day 1,7,14 in BPD infants<br>• FS: significantly higher on day 7, 14, 28 in BPD infants

James et al. *Neonatology* (2015)	47 preterm infants <28 weeks (BPD, n=28)	Supplemental O_2 or respiratory support at 36 weeks PMA	36 weeks PMA	• LV and RV speckle tracking parameters	• Lower RV basal longitudinal strain in BPD vs no BPD infants (mean: –26.4±5.0 vs.–30.7±5.5%, p=0.01)<br>• Lower RV late diastolic strain rate in BPD vs no BPD infants (4.2±1.3 vs. 5.3±1.9, p=0.04)<br>• These associations remained significant (p=0.024 and p=0.015 respectively) after adjustment for gestational age and antenatal steroids.<br>• No difference in RV dimensions nor in LV or septal strain/strain rate.
Levy et al. *J Am Soc Echocardiogr*. (2015)	115 preterm infants <1500 g birth weight (no or mild BPD, n=60; moderate-severe BPD, n=55)	NICHD 2001	Days 1, 3, 32 and 36 weeks PMA	• RV end-diastolic (RVEDD) and end-systolic (RVESD) areas<br>• RV fractional area change (FAC)	• Significantly increased RVEDD and RVSED by 32 weeks PMA in infants with moderate-severe BPD compared to no-mild BPD (p=0.034).<br>• Significantly decreased RV FAC by 32 weeks PMA in infants with moderate-severe BPD compared to no-mild BPD.
Mourani et al. *Am J Respir Crit Care Med* (2015)	274 preterm infants (no BPD, n=50; mild BPD, n=98; moderate BPD, n=92; severe BPD, n=64)	NICHD 2001	7 days and 36 weeks PMA	• Right atrium (RA) enlargement<br>• RV hypertrophy and dilation<br>• IVS flattening<br>• RV systolic pressure (RVSP)<br>• Tricuspid regurgitant jet velocity (TRJV)	• 7 days: higher prevalence of PDA in infants who later developed BPD; no significant difference in other parameters.<br>• 36 weeks PMA: significantly higher prevalence of IVS flattening, RV hypertrophy, RV dilatation in BPD infants (>severe)
Choi et al. *J Cardiovasc Ultrasound* (2016)	73 preterm infants <32 weeks (no BPD, n=28; mild BPD, n=28; moderate BPD,	NICHD 2001	35–37 weeks PMA	• LV EF<br>• Tricuspid regurgitation jet gradient (TRJG)<br>• TDI-MPI	• Mean septal TDI-MPI: significantly higher in the severe BPD group (0.68 ± 0.06) vs. non-BPD (0.58 ± 0.10, p

	n=11; severe BPD, n=6).				<0.01) or mild BPD group (0.59 ± 0.12, p<0.01).<br>• Mean RV TDI-MPI: significantly higher in the severe BPD group (0.71 ± 0.13) than non-BPD group (0.56 ± 0.08, p<0.05) or mild BPD group (0.60 ± 0.125, p<0.05)<br>• Good correlation between BPD severity and RV TDI-MPI (p=0.01, R=0.30) or septal TDI-MPI (p=0.04, R=0.24).
McCrary et al. Am J Perinatol (2016)	88 preterm infants ≤28 weeks (BPD and PH, n=13; BPD only, n=16; controls, n=59).	NICHD 2001 *modified by Ehrenkranz et al.*	>36 weeks PMA (median 38.5-45 weeks)	• LV eccentricity index (EI)<br>• Tricuspid systolic:diastolic (SD) ratio	• No difference in LV-EI and tricuspid SD ratio between the BPD only and the control group
Sehgal et al. *Neonatology* (2016)	28 preterm infants <32 weeks (BPD=18, no BPD=10)	Supplemental O_2 or ventilation support (high-flow O_2, CPAP or mechanical ventilation) at 36 weeks' PMA	36 weeks PMA	• RV FAC<br>• RV TDI parameters and TDI-MPI<br>• Tricuspid annular plane systolic excursion (TAPSE)	• Lower RV-FAC in the BPD vs. no BPD group (31 ± 4.7 vs. 36 ± 3.6%, p=0.019)<br>• Lower TDI peak systolic velocities (0.06 ± 0.01 vs. 0.09 ± 0.01 m/s, p < 0.0001) and peak isovolumic systolic velocities (0.05 ± 0.009 vs. 0.07 ± 0.008 m/s, p<0.0001) in the BPD vs. no BPD group<br>• Higher E/E' ratio in the BPD vs. no BPD group (9.6 ± 1.8 vs. 5.2 ± 0.5, p <0.0001)<br>• Higher RV MPI in the BPD vs. no BPD group (0.33 ± 0.03 vs. 0.25 ± 0.03, p <0.0001)
Haque et al. *J Perinatol* (2017)	34 preterm infants <32 weeks with BPD (mild, n=11; moderate, n=13; severe, n=10) and	NICHD 2001 *modified by Ehrenkranz et al.*	>36 weeks PMA	• TRJV<br>• RV FAC<br>• TAPSE<br>• RV TDI parameters<br>• RV speckle tracking parameters	• Lower free wall middle segment peak systolic strain in severe BPD vs. moderate (p<0.01) and vs. mild/no BPD (p<0.01).

	w/o pulmonary hypertension				• No difference in conventional, RV TDI and in the other speckle tracking parameters.
Seo and Choi. *Cardiovasc Ultrasound* (2017)	67 preterm infants <30 weeks (no BPD, n=22; mild BPD, n=25; moderate BPD, n=4; severe BPD, n=9)	NICHD 2001	Within 14 days and after 28 days of life	• LV EI and FS<br>• TRJV<br>• RV MPI (PW)<br>• TAPSE	• 0-14 days: no association with BPD development.<br>• >28 days: significantly higher RV MPI in severe vs. mild BPD; significantly lower LV-EI in severe vs. no BPD.
Bokiniec et al. *Echocardiography* (2017)[a]	82 preterm infants <32 weeks (no BPD= 32; mild BPD, n=35; severe BPD, n=15).	NICHD 2001	Day 1, 28 and 36 weeks PMA	• RV TDI parameters<br>• RV MPI (PW and TDI)	• Higher RV MPI (PW) in the severe- vs. mild-BPD (p=0.031) and in the severe vs. no BPD group (p=0.014) on day 28
Bokiniec et al. *Echocardiography* (2017)[b]	82 preterm infants <32 weeks (no BPD= 32; mild BPD, n=35; severe BPD, n=15).	NICHD 2001	Day 1, 28 and 36 weeks PMA	• LV TDI parameters<br>• LV MPI (PW and TDI)	• Mean LV E/A (PW) at 36 PMA: significantly lower in the severe vs. mild BPD group (0.73±0.2 vs. 0.94±0.31, p=0.037).<br>• Lower E'-wave velocity in mild vs. no BPD on day 1 (4.32±1.11 vs. 5.62±1.61 cm/s, p=0.006) and day 28 (5.34±1.37 vs. 6.40±1.39 cm/s, p=0.030); no difference between severe and mild or no BPD.<br>• Lower E/E' ratio (PW): in mild vs. no BPD group on day 1 (9.03±2.56 vs. 7.21±1.85, p=0.019).
Ehrmann et al. *J Pediatr* (2018)	93 preterm infants <30 weeks (no BPD, n=47; BPD, n=46)	NICHD 2001	36 weeks PMA	• LV EI<br>• PAAT and PAAT:ET ratio<br>• RV FAC and systolic to diastolic ratio<br>• TAPSE<br>• RV TDI-MPI<br>• RV speckle tracking parameters	• Higher EI in the BPD group (OR 1.49, (95% CI 1.13 – 2.12, p = 0.01).<br>• No difference in the other ECHO parameters.

Levy et al. *J Pediatr* (2018)	80 preterm infants <29 weeks (BPD, n=48)	NICHD 2001 *modified by Poindexter et al.*	1 year CA	• RV FAC<br>• PAAT and PAAT:ET ratio<br>• RV and LV speckle tracking parameters	• Lower RV FAC in BPD compared to no BPD infants (30±4 vs. 34±3, p<0.01)<br>• Lower PAAT (69±5 vs. 79±4) and PAAT:ET ratio (0.28±0.06 vs. 0.31±0.04) in BPD compared to no BPD infants.<br>• Lower RV free wall longitudinal strain (FWLS) in BPD compared to no BPD infants (−23±3 vs.−25±1, p<0.01); no difference in LV FWLS.
Alvarez-Fuente et al. *Plos One (*2019)	47 preterm infants <28 weeks (no/mild BPD n=25, moderate/severe BPD n=22)	NICHD 2001	Days 3, 7, 14, 21, 28	IVS flattening	IVS flattening: OR 3.63 for predicting moderate or severe BPD
Erickson et al. *Cardiol Young* (2019)	80 preterm infants <29 weeks (BPD, n=48; no BPD, n=32)	NICHD 2001 *modified by Poindexter et al.*	32 weeks PMA and 1 year CA	• RV FAC<br>• PAAT and PAAT:ET ratio<br>• RV speckle tracking parameters (FWLS, FWLS rate, basal, mid-ventricular and apical longitudinal strain).	• Lower RV FAC in BPD vs. no BPD infants at 32 weeks PMA (median 30 [IQR 26- 33] vs. 36 [32-43] %, p<0.01) and 1 year CA (33 [31-34] vs. 39 [36-43] %, p<0.01).<br>• Lower TAPSE in BPD vs. no BPD infants at 32 weeks PMA (8 [7-9] vs. 9 [8- 10], p<0.01) and 1 year CA (16 [15-17] vs. 18 [16-19], p<0.01).<br>• Lower magnitudes of FWLS, FWLS rate and basal, mid-ventricular and apical longitudinal strain at 32 weeks PMA (p<0.01 for all parameters) and 1 year CA (p<0.01 for all parameters)<br>• Lower PAAT in BPD vs. no BPD infants at 32 weeks PMA (43 [35- 53] vs. 56 [45-66]

					msec, p<0.01) and 1 year CA (65 [60-69] vs. 79 [69-82] msec, p<0.01).<br>• Lower PAAT:ET ratio in BPD vs. no BPD infants at 32 weeks PMA (0.23 [0.20, 0.27] vs. 0.31 [0.23-0.36], p<0.01) and 1 year CA (0.27 [0.25- 0.29] vs. 0.31 [0.30-0.36], p<0.01).
Patel et al. *J Am Soc Echocardiogr* (2019)	222 preterm infants <29 weeks (BPD, n=116; control group, n=103)	NICHD 2001 *modified by Poindexter et al.*	Days 1, 2, 5–7, 32 and 36 weeks PMA, 1 year CA	• PAAT and PAAT:ET ratio	• Lower PAAT and PAAT:ET ratio in BPD vs. no BPD infants on day 2 (44±8 vs. 47±6 msec, p=0.005 and 0.25±0.02 vs. 0.27±0.01, p=0.01, respectively), 32 weeks PMA (p<0.01), 36 weeks PMA (p<0.001) and 1 year CA (p<0.001).<br>• PAAT differences persisted after adjustment for surfactant, antenatal and postnatal steroids.
Lehmann et al. *Cardiol Young* (2020)	117 preterm infants <28 weeks (BPD, n=69; PH=16; PDA, n=25 at 32 weeks PMA, n=14 at 36 weeks PMA)<br><br>*Merged group “cardiopulmonary complications”: BPD or PH or PDA*	NICHD 2001 *modified by Poindexter et al.*	32 and 36 weeks PMA, 1 year CA	• LV speckle tracking parameters	• Decrease in torsion from 32 to 36 weeks PMA, but not later, differed significantly between “complicated” infants and controls (6.62±2.85 °/cm to 5.07±2.02 °/cm vs. 5.37±2.35 °/cm to 5.32±2.14°/cm, p=0.019)<br>• No significant difference in the torsion changes from 36 weeks PMA to 1 year CA<br>• No between-group difference at 1 year CA
Mendez-Abad et al. *Front Ped* (2020)	101 preterm infants <32 weeks and/or <1500g<br>(no BPD, n=86; BPD, n=15).	NICHD 2001	Days 1, 3 and weekly until 36 weeks PMA	• TAPSE<br>• RV TDI parameters	• Reduced TAPSE in the BPD group (B=−6.68, 95% CI −13.14 to −0.23, p=0.042)

					• Lower A' wave in the BPD group (B=−0.64, 95% CI −1.20 to −0.087, p=0.023)
Vyas-Read et al. *J Perinatol* (2020)	1157 preterm infants <32 weeks with severe BPD (sBPD), of with 115 died.<br><br>*Retrospective study*	Severe BPD: positive pressure ventilation, >2 L/min by NC, or FiO_2 >0.3 at ≥ 36 weeks PMA	34-44 weeks PMA	• IVS position (IVSP)<br>• RVSP	• Abnormal IVSP: increased mortality rates (19% vs. 8%, p <0.01) in unadjusted and adjusted multivariable model (SP OR 1.9, 95% CI 1.2–3.0)<br>• Abnormal RVSP: increased mortality (20% vs. 9%, p <0.01) in unadjusted and adjusted multivariable model (RVSP OR 2.2, 95% CI 1.1–4.7)
Aldana-Aguirre et al. *Echocardiography* (2021)	58 extremely preterm infants (no BPD, n=38; BPD, n=20).	NICHD 2001	Day 28 and 37 weeks PMA	• LV FS<br>• LA/Ao ratio<br>• LVO and RVO<br>• RV FAC<br>• RV ET<br>• PAAT and PAAT:ET ratio<br>• TAPSE<br>• RV and LV TDI parameters<br>• RV and LV speckle tracking parameters	• 28 days ECHO: higher LVO, lower tricuspidal e', a' and s' velocities, higher E/e' ratio, reduced LV apical circumferential early diastolic strain rate, twist rate, untwist rate and torsion, reduced RV systolic strain rate in BPD vs. no BPD infants<br>• 37 weeks' PMA ECHO: higher LV FS and LA/Ao ratio, lower PAAT, lower tricuspidal e', a' and s' and septal e' velocities, higher E/e' ratio, increased LV late diastolic strain rate and reduced LV untwist rate in BPD vs. no BPD infants
Neumann et al. *Plos One* (2021)	294 very preterm infants (BPD, n=57; died, n=10)*<br><br>**merged outcome (BPD or death)*	Supplemental O_2 at 36 weeks PMA	Day 7	• TAPSE<br>• RV MPI (PW)	• TAPSE: significantly decreased in the BPD/death group vs. controls (5 [5-6] vs 6 [5.4-7] mm).<br>• RV MPI (PW): increased in the BPD/death group vs. controls (median [IQR] 0.3 [0.23-0.38] vs 0.22 [0.15-0.29]); independent predictor of

BPD/death (β=4.112, p=0.012) at multivariate analysis.
