# Supplementary material for: A scoping review of echocardiographic and lung ultrasound biomarkers of bronchopulmonary dysplasia in preterm infants
Source: Front Pediatr. 2023 Feb 10;11:1067323. doi: 10.3389/fped.2023.1067323 (PMC9950276; doi:10.3389/fped.2023.1067323)
Supplement: Supplementary file 2 [file Datasheet2.pdf]

Supplemental Table 2. Details of clinical studies investigating echocardiographic parameters for pulmonary hypertension (PH) assessment or prediction, listed in chronological order by year of publication.

Author	Study sample	PH/BPD definition	ECHO timing	ECHO parameters	Main findings
Abraham et al. *Echocardiography* (2016)	207 preterm infants at risk for PH (birth weight <750 g or <32 weeks with BPD diagnosis)<br><br>*Retrospective study*	BPD: NICHD 2001<br><br>PH: presence of systolic IVS flattening or TRJG >36 mmHg or RVSP >50% of systemic arterial blood pressure (sABP) at ECHO (see ECHO timing)	Mean age of 76±20 days	• Left ventricular eccentricity index (LV-EI) (<1.15, 1.15–1.29, and≥1.3)	• Systolic IVH flattening was evident at LV-EI≥1.15<br>• Half-systemic RVSP became apparent at LV-EI≥1.3<br>• Quantitative parameters of RV systolic function were impaired only at LV-EI≥1.3
McCrary et al. Am J Perinatol (2016)	88 preterm infants ≤28 weeks (BPD and PH, n=13; BPD only, n=16; controls, n=59).	NICHD 2001 *modified by Ehrenkranz et al.*<br><br>PH defined by cardiac catheterization data, peak TRJV, and/or clinical need/response to pulmonary vasodilators.	>36 weeks PMA (median 38.5-45 weeks)	• LV-EI<br>• Tricuspid systolic:diastolic (SD) ratio	• Higher LV-EI in infants with BPD and PH compared to those with BPD only and no BPD (systolic LV-EI: 1.46 vs 1.00–1.01, p≤0.01 for both comparisons; diastolic LV-EI: 1.47 vs 1.00-1.00, p≤0.01 for both comparisons).<br>• Higher tricuspid SD ratio in infants with BPD and PH compared to those with BPD only and no BPD (1.12 vs 0.97–1.00, p≤0.01 for both comparisons).
Richardson et al. *Pediatr Cardiol* (2016)	Preterm infants <35 weeks (PH, n=22)	PH: RVSP >50% of sABP, any shunt with bidirectional or right-to-left flow, flattening or posterior bowing of IVS at ECHO (see ECHO timing)	Median age: 66 postnatal days	• Tricuspid annular plane systolic excursion (TAPSE)<br>• LV and RV TDI parameters	• Lower TAPSE (median 0.70 [IQR 0.50–0.99] vs. 0.95 [0.72–1.50] cm, p=0.034) in PH infants vs. controls.<br>• Lower mitral S' (4.84 [4.28–5.75] vs. 5.71 [4.92–6.65], p=0.041) and E' (5.37 [4.24–6.50] vs. 7.80 [5.86–9.74], p=0.044) PH infants vs. controls.

					• Lower basal IVS S' (4.55 [4.05–5.68] vs. 5.55 [4.54–6.65] p=0.004) and E' (4.73 [3.20–6.03] vs. 6.12 [4.38–8.17], p=0.044) in PH infants vs. controls.<br>• Lower lateral tricuspid S' (7.13 [5.77–8.02] vs. 7.89 [7.31–10.13], p=0.020) and E' (7.76 [4.81–10.07] vs. 9.19 [7.10–12.27], p=0.013) in PH infants vs. controls.
Levy et al. *J Am Soc Echocardiogr* (2017)	239 extremely preterm infants (PH, n=35).	BPD: supplemental O_2 at 36 weeks' PMA<br><br>~~Late~~ PH: RVSP >40mmHg or >50% of sABP, any shunt with bidirectional or right-to-left flow, unusual degree of RV hypertrophy, dilatation or IVS flattening at any ECHO performed between 32 weeks PMA and 1 year CA (see ECHO timing).	Days 1, 2, 5-7, 32 and 36 weeks PMA, 1 year CA	LV and right ventricle (RV) speckle tracking parameters:<br>• Free wall longitudinal strain (FWLS) and strain rate (FWLSRs)<br>• Longitudinal strain (LS) and strain rate (LSR)<br>• Global longitudinal strain (GLS) and strain rate (GLSR)<br>• Segmental longitudinal strain (SLS)<br>• Systolic strain rate (SSR)	• Persistent base-to-apex gradient at days 5-7 associated with PH at 36 weeks PMA (RR 2.15; 95%CI, 1.18–4.33; p=0.02).<br>• Decreased RV FWLS (p=0.01) and IVS GLS (p=0.002) at 32 and 36 weeks PMA in infants with PH persistence at 1 year CA vs. uncomplicated infants; no difference in LV GLS, GLSR and FW SLS patterns.<br>• Altered IVS base-to-apex SLS gradient at 1 year CA even after adjustment for BPD (b=2.9, p<0.001)
Levy et al. *J Pediatr* (2018)	80 preterm infants <29 weeks (late PH, n=12)	NICHD 2001 *modified by Poindexter et al.*<br><br>Late PH: RVSP >40mmHg or >50% of sABP, any shunt with bidirectional or right-to-left flow, moderate-	1 year CA	• RV fractional area change (FAC)<br>• Pulmonary artery acceleration time (PAAT) and PAAT to right ventricular ejection time ratio (PAAT:ET)	• Lower RV FAC in late PH infants compared to controls (30±4 vs. 34±3, p<0.01)<br>• Lower PAAT (70±5 vs. 74±4) and PAAT:ET ratio (0.29±0.03 vs. 0.32±0.03) late PH infants compared to controls.<br>• Lower RV free wall longitudinal strain (FWLS) late

		severe IVS flattening at 12 months CA ECHO		• RV and LV speckle tracking parameters	PH infants compared to controls (−22±1 vs.−26±1, p<0.01); no difference in LV FWLS.
Behere et al. *Pediatr Cardiol* (2019)	37 preterm infants with BPD (early PH, n=9; late PH, n=5).	BPD: supplemental O_2 or PPV at 36 weeks' PMA/discharge if <32 weeks (or at 56 days/discharge if ≥32 weeks)<br><br>Early PH: IVS flattening and/or RVSP >50% of sABP (see ECHO timing)<br><br>• Early PH: criteria met at initial ECHO (see timing)<br>• Late PH: criteria met at last follow-up ECHO (see timing)	Early PH: 39.0±2.9 weeks PMA<br><br>Late PH: 61.5±62.4 weeks PMA	• RA enlargement<br>• RV hypertrophy<br>• Interventricular septum (IVS) flattening<br>• RV end-diastolic and end systolic areas<br>• RV FAC<br>• RV systolic pressure (RVSP)<br>• RV systolic time interval (STI)<br>• Main pulmonary artery (MPA) diameter (z-score)<br>• RV/LV ratio<br>• RV TDI parameters and TAPSE	*Early PH vs. no early PH:*<br>• Higher rates of RA dilation and RV hypertrophy (p<0.001 both)<br>• Higher RVSP (49.2±16.8 vs. 23.7±2.1, p=0.03)<br>• Increased end-diastolic (4.2 ± 1.7 vs. 2.8 ± 0.8, p=0.03) and end-systolic areas (2.9 ± 1.3 vs. 1.8 ± 0.4, p=0.03) but no difference in RV-FAC<br><br>*Late PH vs. no late PH:*<br>• Higher rates of RA, dilation (p=0.01) and RV hypertrophy (p=0.004)<br>• Lower RV FAC (27.2±6.8 vs. 36.9±6.5, p=0.03)<br>• Higher MPA diameter z-scores (1.9±0.7 vs. 1.2±0.9, p=0.02)
Blanca et al. *Pulm Circ* (2019)	69 preterm infants with severe BPD (PH, n=8; no PH, n=61).	Severe BPD: supplemental O_2 for ≥ 28 days and need for either >30% O_2, >1 L/min flow, CPAP or ventilator support at 36 weeks PMA<br><br>PH: TRJV >2.8 m/s and/or flat or leftward deviated IVS at 6 months CA ECHO	6 months CA	• LV fractional shortening (FS)<br>• RVSP<br>• RV FAC<br>• PAAT<br>• RV and LV speckle tracking parameters	• Higher RVSP in PH group compared to no PH (33±3.7 vs. 18.1±3.6, p<0.001)<br>• Reduced FAC in PH group compared to no PH (35±9 vs 43±9, p=0.03)<br>• Reduced RV GLSR (p=0.04)
Patel et al. *J Am Soc Echocardiogr* (2019)	222 preterm infants <29 weeks (PH, n=17; controls, n=103)	Late PH: RVSP >40mmHg or >50% of sABP, any shunt with	Days 1, 2, 5–7, 32 and 36	• PAAT and PAAT:ET ratio	• No difference in PAAT and PAAT:ET ratio between infants

		bidirectional or right-to-left flow, unusual degree of right ventricular hypertrophy or dilatation, or IVS flattening at aby ECHO beyond day 5-7 through 1 year CA	weeks PMA, 1 year CA		with and without PH from day 1 to 7.<br>• Decreased PAAT and PAAT:ET ratio in PH compared to control infants at 32 and 36 weeks PMA (p <0.001 at both time points) even after adjustment for BPD (b= 2.1, p=0.002).<br>• Lower PAAT and PAAT:ET ratio at 1 year CA in infants with PH at 32 and/or 36 weeks PMA compared to controls.<br>• A PAAT <47 msec and PAAT:ET ratio <0.28 at 32 weeks PMA resulted in sensitivity of 91% and specificity of 95% for PH detection at 36 weeks PMA (AUC 0.93, 95% CI 0.88–0.97).
Nawaytou et al. *J Am Soc Echocardiogr* (2020)	29 preterm infants (<30 weeks at birth) undergoing cardiac catheterization<br><br>*Retrospective study*	PH (ECHO): TRJV >2.9 m/sec or RVSP >35 mm Hg, or evidence of IVS flattening<br><br>PH (cardiac catheterization): BSA-indexed PVR ≥3WU, mean pulmonary artery pressure > 20mmHg and pulmonary artery wedge pressure ≤15mmHg at cardiac catheterization.	Median age of 49 weeks' PMA<br><br>Median period between ECHO and cardiac catheterization: 1 day	• RV/LV ratio<br>• PAAT<br>• RV MPI<br>• RV systolic to diastolic time index (SDI, measured by TDI)	In the absence of post-tricuspid valve shunts, a significant correlation was observed between PVR (measured by cardiac catheterization) and:<br>• RV MPI (ρ= 0.89, p=0.005)<br>• RV SDI (ρ=0.84, p<0.001)<br>• RV/LV end systolic diameter ratio (ρ=0.66, p=0.003)<br>• PAAT (ρ=0.48, p=0.05).
Savoia et al. *Eur J Pediatr* (2021)	23 preterm infants <32 weeks or ≤ 1500g with moderate or severe BPD (mild PH, n=12)	BPD: NICHD 2001<br><br>PH: RVSP >50% of sABP or evidence of	36 weeks PMA; 2, 4, 6, 8 months postnatal age.	• LV FS<br>• RV, LV and IVS diastolic diameters	• Lower PAAT (36 weeks: 68.9 [interquartile range, IQR: 11.9] vs. 52.0 [19.1], p=0.044; 6 months: 83.9 [38.9] vs 74.8

	Retrospective study	IVS flattening at any ECHO<br><br>Mild PH: RVSP <50% sABP or mild septal flattening at any ECHO		• RV/LV ratio<br>• PAAT and PAAT/ET ratio<br>• LV and RV TDI parameters	[16.9], p=0.037) in PH vs. no PH infants.<br>• Lower PAAT/ET ratio (36 weeks: 0.24 [IQR: 0.08] vs. 0.24 [0.08], p=0.031; 6 months: 0.35 [IQR: 0.04] vs. 0.37 [0.08], p=0.005) in PH vs. no PH infants.<br>• No difference in the other parameters.
Madden et al. *Pediatr Cardiol* (2022)	64 preterm infants with severe BPD (PH, n=22)<br><br>*Retrospective study*	BPD: NICHD 2001<br><br>PH: IVS flattening in systole and/or RVSP >50% of sABP and/or evidence of right-to-left or bidirectional shunts at 36 weeks PMA ECHO	36 weeks PMA	• RA dilation<br>• RV hypertrophy and dilation<br>• RV FAC<br>• RVSP<br>• MPA diameter (z-score)<br>• LV-EI	*Predictors of PH at discharge:*<br>• RV hypertrophy (OR 4.6)<br>• RV dilation (OR 3.1)<br>• Reduced diastolic EI (OR 1.46)<br>• Increased MPA z-score (OR 1.90)<br>• RV FAC <30.4 % (sensitivity 0.73, specificity 0.82)
Sallmon et al. *J Perinatol* (2022)	34 extremely preterm infants (BPD without PH, n=19; BPD with PH, n=15)	PH: TRJV >2.5m/s in the absence of RV outflow tract obstruction at 3 months (chronological age) ECHO	3 and 12 months postnatal age	• RVSP<br>• TAPSE and TAPSE/RVSP ratio<br>• PAAT<br>• RV/LV ratio<br>• LV-EI	• Higher RVSP in PH vs. no PH infants at 3 (34 [IQR 30-41] vs. 21 [19-23] mmHg, p<0.001) and 12 months (30.5 [28–34) vs. 21 (19-22) mmHg, p<0.001).<br>• Lower TAPSE in PH vs. no PH infants at 3 (6.6 [6-8] vs. 9 [7.5-9] mm, p=0.005) and 12 months (8.8 [7.8-10.3] vs. 13.8 (12.6-14.7) mm, p<0.001)<br>• Lower TAPSE/RVSP ratio in PH vs. no PH infants at 3 (0.22 [0.17-0.23] vs. 0.37 [0.33-0.47], p<0.001) and 12 months (0.32 [0.26-0.41) vs. 0.62 [0.52-0.72], p<0.001)<br>• Lower PAAT in PH vs no PH infants at 3 (45 [40–51] vs. 60 [53–65] ms, p<0.001) and 12

	months (61 [53-66) vs. 83 [77–88] ms, p<0.001]<br>• Higher LV-EI in PH vs. no PH infants at 3 (1.4 [1.32–1.5] vs. 1.06 [1-1.19], p<0.001) and 12 months (1.23 [1.18-1.3] vs. 1.03 [1-1.1], p<0.001)<br>• Increased RV/LV end-systolic ratio in BPD vs no PH infants at 3 (1.09 [1.05-1.15] vs. 0.9 [0.82-0.93], p<0.001) and 12 months (1.02 [0.98-1.07] vs. 0.87 [0.85–0.94], p<0.001)
