# Supplementary material for: A scoping review of echocardiographic and lung ultrasound biomarkers of bronchopulmonary dysplasia in preterm infants
Source: Front Pediatr. 2023 Feb 10;11:1067323. doi: 10.3389/fped.2023.1067323 (PMC9950276; doi:10.3389/fped.2023.1067323)
Supplement: Supplementary file 3 [file Datasheet3.pdf]

Supplemental Table 3. Details of clinical studies investigating lung ultrasound scores and the related cut-off proposed for BPD prediction, listed in chronological order by year of publication. Modified with permission from Corsini et al (31). Abbreviations: BPD: bronchopulmonary dysplasia; LUS: lung ultrasound score; CI: confidence interval; AUROC: Area under the receiver operating characteristic curve; PMA: post menstrual age.

Author	Study sample	BPD definition	LUS protocol	LUS timing	Cut-off for BPD prediction	Sensitivity,% (95% CI)	Specificity,% (95% CI)	AUROC (95% CI)
Abdelmawla et al. *Am J Perinatol* (2019)	27 preterm infants <32 weeks (BPD, n=14)	Need of positive pressure support or supplemental oxygen therapy at 36 weeks PMA	8-areas (4 per hemithorax: upper anterior, lower anterior, lateral, and costophrenic angle for assessment of pleural effusions)	2–8 weeks	≥6	76	97	0.94 (0.78–0.97)
Alonso-Ojembarrena et al. *Pediatr Pulmonol* (2019)	59 preterm infants ≤ 1500g and/or ≤32 weeks (BPD, n=21)	Walsh 2004	6-areas (3 per hemithorax: upper anterior, lower anterior and lateral)	Day 7	≥5	71 (47-87)	79 (59-87)	0.8 (0.65-0.91)
				Day 14	≥5	74 (51-88)	100 (82-100)	0.93 (0.8-0.99)
				Day 28	≥4	100 (65-100)	78 (61-89)	0.89 (0.75-0.97)
Hoshino et al. *Am J Perinatol* (2020)	87 preterm infants <32 weeks (mild BPD, n=39; moderate BPD, n 33; severe BPD, n=15)	NICHD 2001	6-areas (3 per hemithorax: anterior, lateral and posterior)	Day 28	≥ 7	85 (72–94)	92 (79–98)	0.95 (0.91-0.99)
Oulego-Erroz et al. *J Perinatol* (2020)	42 preterm infants <32 weeks (no BPD, n=21; mild BPD, n=8; moderate BPD, n=9, severe BPD, n=4)	NICHD 2001	8-areas (4 per hemithorax: upper anterior, lower anterolateral, lower posterolateral, and lower posterior)	Day 7	≥ 8	93	91	0.94 (0.87–1)
				Day 7	≥ 5	90	81	0.93 (0.86–1)
Liu X et al. *Eur J Pediatr* (2021)	130 preterm infants < 32 weeks (no BPD, n=80; mild BPD, n=16; moderate	Jensen 2019, any grade	10-areas (5 per hemithorax: upper anterior, lower anterior, lateral, upper posterior, and lower posterior)	Day 12	>8	72 (57.5-83.8)	85.9 (76.2-92.7)	0.832 (0.756–0.892)

	BPD, n=23; severe BPD, n=11)		12-areas (6 per hemithorax: upper anterior, lower anterior, upper lateral, lower lateral, upper posterior, and lower posterior)	Day 12	>8	74 (59.7–85.4)	84.6 (74.7-91.8)	0.834 (0.758–0.894)
Mohamed et al. *J Pediatr* (2021)	152 extremely preterm infants (no BPD, n=65; mild BPD, n=65; moderate BPD, n=35; severe BPD, n=3)	Need of supplemental O_2 or respiratory support at 36 weeks of PMA or at discharge, if earlier	6-areas (3 per hemithorax: upper anterior, lower anterior and lateral)	Day 3	>11	95 (90-100)	80 (72-89)	0.964 (0.94-0.987)
				Day 7	>10	89 (82- 97)	90 (83-96)	0.966 (0.943-0.989)
				Day 14	>11	91 (84-98)	83 (75-91)	0.948 (0.918-0.977)
Alonso-Ojembarrena et al. *Chest* (2021)	298 preterm infants <32 weeks (moderate-severe BPD, n=73)	Walsh 2004, moderate to severe	6-areas (3 per hemithorax: upper anterior, lower anterior and lateral)	Day 3	≥7	80 (58-92)	71 (61-79)	0.77 (0.68-0.85)
				Day 7	≥8	70 (58-80)	79 (72-84)	0.79 (0.74-0.84)
				Day 21	≥7	77 (59-88)	74 (65-81)	0.80 (0.72- 0.86)
Aldecoa-Bilbao et al. *Neonatology* (2021)	89 preterm infants <32 weeks (BPD NICHD 2001 n=41;BPD Jensen, n=23)	NICHD 2001, any grade	6-areas (3 per hemithorax: mid-clavicular line, anterior axillary line, posterior axillary line)	Day 7	≥8	70 (55–80)	91 (79–96)	0.87 (0.79–0.94)
		Jensen 2019, any grade	6-areas (3 per hemithorax: mid-clavicular line, anterior axillary line, posterior axillary line)	Day 7	≥9	65 (45–81)	82 (71–89)	0.80 (0.70–0.90)
Loi et al (2021)	147 preterm infants < 31 weeks (BPD, n=72)	NICHD 2001	6-areas (3 per hemithorax: upper anterior, lower anterior and lateral)	Day 7	0,23 (adjusted LUS: LUS/GA)	71 (59-81)	74 (63-84)	0.826 (0.742-0.891)
			10-areas (4 per hemithorax: upper anterior, lower anterior lateral, upper posterior and lower posterior)	Day 7	0.43 (adjusted LUS: LUS/GA)	86 (74-94)	65 (51-77)	0.833 (0.745-0.893)
			6-areas (3 per hemithorax: upper anterior, lower anterior and lateral)	Day 14	0.31	66 (59-82)	81 (70-89)	0.834 (0.751-0.898)

					(adjusted LUS: LUS/GA)			
			10-areas (4 per hemithorax: upper anterior, lower anterior, lateral, upper posterior and lower posterior)	Day 14	0.59 (adjusted LUS: LUS/GA)	76 (63-87)	84 (72-93)	0.858 (0.780-0.917)
Raimondi et al. *Pediatrics* (2021)	240 preterm infants <34 weeks (BPD, n=23)	Jensen 2019, any grade	6-areas (3 per hemithorax: mid-clavicular line, anterior axillary line, posterior axillary line)	Day 7	≥10	68	82	0.82 (0.71-0.93)
Martini et al. *Eur J Pediatr* (2022)	64 preterm infants ≤34 weeks (BPD, n=17)	NICHD 2001	6-areas (3 per hemithorax: mid-clavicular line, anterior axillary line, posterior axillary line)	Days 1,2,3	≥9	75.5	70.4	0.77 (0.68–0.84)
